# Amplifying Anti‐Tumor Immune Responses via Mitochondria‐Targeting Near‐Infrared Photodynamic Therapy

**DOI:** 10.1002/advs.202505525

**Published:** 2025-06-09

**Authors:** Cheng‐Ao Li, Junjie Nan, Qingxuan Ye, Bingzhu Zheng, Xiaomeng Dai, Jingya Li, Feng Wang, Huimin Ma, Yu Cheng, Jian Ruan, Weijia Fang, Peng Zhao, Renren Deng, Dong Cen

**Affiliations:** ^1^ State Key Laboratory of Silicon and Advanced Semiconductor Materials Institute for Composites Science Innovation School of Materials Science and Engineering Zhejiang University Hangzhou 310058 China; ^2^ Department of Medical Oncology The First Affiliated Hospital School of Medicine Zhejiang University Hangzhou 310003 China; ^3^ Department of General Surgery Sir Run Run Shaw Hospital School of Medicine Zhejiang Key Laboratory of Minimally Invasive Technique and Precision Medicine Zhejiang University Hangzhou 310016 China; ^4^ State Key Laboratory of Baiyunobo Rare Earth Resource Researches and Comprehensive Utilization Baotou Research Institute of Rare Earths Baotou 014030 China

**Keywords:** anti‐tumor immunity, near‐infrared, orthotopic liver tumor, photodynamic therapy

## Abstract

Pro‐inflammatory photodynamic therapy (PDT) holds immense potential to ignite robust and long‐lasting systemic anti‐tumor immune responses. However, the limited penetration depth of conventional ultra violet (UV)‐visible irradiation and the tumor hypoxia microenvironment significantly constrain the efficacy of immune‐regulatory PDT. Here, a mitochondria‐targeting enhanced nanoplatform (NZ_@TG_) is reported, activated by near‐infrared (NIR) light‐driven PDT, to address these challenges and amplify systemic anti‐tumor immunity. This nanoplatform employs an interfacial lanthanide‐organic triplet photosensation mechanism to realize localized oxidative damage of oxygen‐rich mitochondria under NIR irradiation. Simultaneously, the exacerbated hypoxia induced by PDT activates a TH302 prodrug, resulting in cell cycle arrest in highly proliferative tumor cells. These combined effects trigger immunogenic cell death (ICD), releasing damage‐associated molecular patterns (DAMPs) to activate immune responses. This approach demonstrates significantly enhanced tumor ablation in deep‐seated lesions in orthotopic liver tumor models and induces long‐term anti‐tumor immune memory. Moreover, the NIR‐PDT‐induced immune activation markedly improves the immune checkpoint blockade (ICB) therapy efficacy. This strategy offers a robust modality for immune activation in cancer therapy, paving the way for effective treatment of deep‐seated tumors and preventing recurrence.

## Graphical abstract

Interfacial Nd^3+^‐triplet energy transfer enables direct activation of molecular triplets under near‐infrared (NIR) irradiation for reactive oxygen species (ROS) generation. Photodynamic therapy (PDT) exaggerates hypoxia, which initiates subsequent hypoxia‐activated chemotherapy. Synergistically, immunogenic cell death is induced to amplify immune responses. The immunostimulatory NIR‐PDT nanoplatform exhibits remarkable anti‐tumor efficacy in deep‐seated orthotopic liver tumor models.

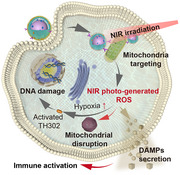

## 1 Introduction

Photodynamic therapy (PDT) employs non‐toxic photosensitizers (PSs) to produce cytotoxic reactive oxygen species (ROS) under light irradiation, enabling direct cell ablation on tumor lesions in a spatiotemporally controllable manner. Apart from killing cancer cells instantly under light irradiation, PDT offers a unique advantage of immune activation in eliciting long‐term anti‐tumor effects against metastasis and recurrence,^[^ ^1^, ^2^, ^3^, ^4^ ^]^ because of its ability to reshape the tumor microenvironment and the surrounding vasculature through photodynamic priming (PDP).^[^ ^5^ ^]^ Specifically, PDT‐initiated oxidative damage can trigger immunogenic cell death (ICD), featuring the release of damage associated molecular patterns (DAMPs) that promotes dendritic cell maturation, macrophage polarization, and antigen presentation to cytotoxic T cells. Despite the promising, PDT faces significant challenges in clinical applications, primarily due to the limited light penetration depth and the hypoxia nature of tumor microenvironments.

Most organic PSs for PDT, such as curcumins, porphyrins, and phthalocyanines, are activated by ultra violet (UV)‐Vis light, which suffer from strong absorption and scattering by biological tissues, limiting its therapeutic efficacy to only superficial diseases.^[^ ^6^, ^7^, ^8^, ^9^ ^]^ In contrast, near‐infrared (NIR) light offers substantially deeper tissue penetration owing to suppressed light absorption and scattering, making it a more suitable alternative for PDT in deep‐seated tumors.^[^ ^10^, ^11^, ^12^ ^]^ While NIR‐responsive organic PSs have been developed, challenges such as photostability, biocompatibility, and complex molecular synthesis remain to be tackled. In recent years, a series of inorganic nanomaterial‐based PSs with NIR responsiveness have played an important role in mitigating the penetration dilemma in PDT research.^[^ ^13^, ^14^, ^15^, ^16^, ^17^, ^18^ ^]^ An alternative promising strategy involves integrating photo‐stable lanthanide‐based nanomaterials with organic PSs, leveraging the NIR responsiveness of lanthanides such as Nd^3+^ and Yb^3+^ to enhance NIR light‐driven PDT.^[^ ^19^, ^20^, ^21^, ^22^ ^]^ Recently, NIR‐activated lanthanide‐based nanomaterials have also been employed for real‐time imaging guided phototherapy,^[^ ^23^, ^24^, ^25^ ^]^ and for synergistic treatment approaches with immunotherapy,^[^ ^26^, ^27^ ^]^ significantly expanding its applications in cancer diagnostics and therapeutics.

Tumor hypoxia leads to insufficient supply of O_2_ for singlet oxygen (^1^O_2_) generation in type II PDT process. Countermeasures have been taken such as developing type I PSs with less O_2_‐dependency or catalyzing overexpressed H_2_O_2_ in tumors for in situ O_2_ production. Inside cells, mitochondria serve as the central organelles for cellular metabolism and oxygen consumption, thus rendering their pivotal role in dynamic therapeutic research.^[^ ^15^, ^28^ ^]^ Inhibiting physiological functions of mitochondria has been proven to be a rational method to save intracellular oxygen for PDT.^[^ ^29^, ^30^ ^]^ In this regard, targeting PDT agents to mitochondria may mitigate the hypoxia limitation in the tumor microenvironment by localizing ROS generation near oxygen‐rich sites. Mitochondrial disruption by ROS can further amplify oxidative stress, leading to cellular apoptosis. Additionally, mitochondria are intricately linked to immune responses, as they are greatly involved in cell proliferation, apoptosis, calcium homeostasis, and so on.^[^ ^31^, ^32^ ^]^ Thus, we propose that mitochondria‐targeting PDT has the potential to not only enhance ROS‐mediated tumor cell death but also potentiate systemic anti‐tumor immune responses.

**Scheme 1:**
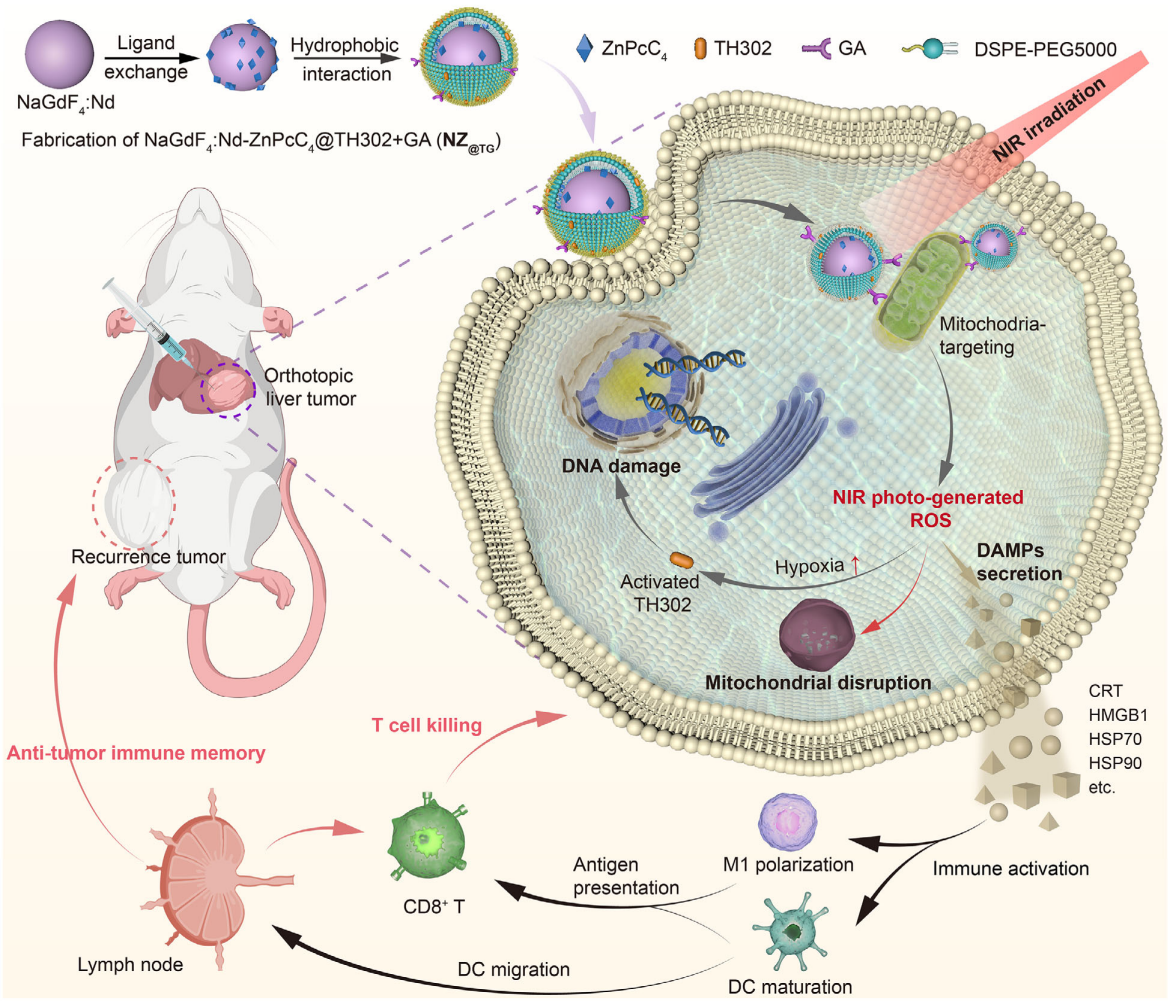
Design of NZ_@TG_ nanocomposites for cancer therapy and schematic illustration of the anti‐tumor mechanisms via near infrared irradiation triggered oxidative stress, DNA damage, and mitochondrial disruption that induce ICD and activate systemic immune responses. Schematic illustration was created with BioGDP (https://biogdp.com/).

Herein, we report a mitochondria‐targeting nanoplatform (NZ_@TG_) that combines NIR‐responsive PDT with hypoxia activated chemotherapy to address the limitations of conventional PDT approaches (**Scheme** **1**). The nanoplatform integrates a lanthanide‐doped core nanocrystal (NaGdF_4_:Nd) with a zinc tetracarboxyphthalocyanine (ZnPcC_4_) organic photosensitizer for efficient ROS generation under NIR irradiation. Functionalized with glycyrrhetinic acid (GA) for mitochondrial targeting and loaded with the hypoxia‐activated prodrug TH302, NZ_@TG_ simultaneously induces oxidative damage and hypoxia‐mediated DNA impairment. This dual action triggers ICD, releasing DAMPs that activate systemic anti‐tumor immune responses. Our study demonstrates the therapeutic efficacy of NZ_@TG_ in both subcutaneous and orthotopic liver tumor models, highlighting its potential for deep‐tissue cancer treatments. Furthermore, NZ_@TG_‐initiated immune activation significantly enhances immune checkpoint blockade (ICB) therapy efficacy, providing a synergistic strategy for malignant cancer immunotherapy.

## 2 Results and Discussion

### 2.1 Singlet Oxygen Generation of the NZ_@TG_ Nanoplatform under NIR Irradiation

**Figure 1:**
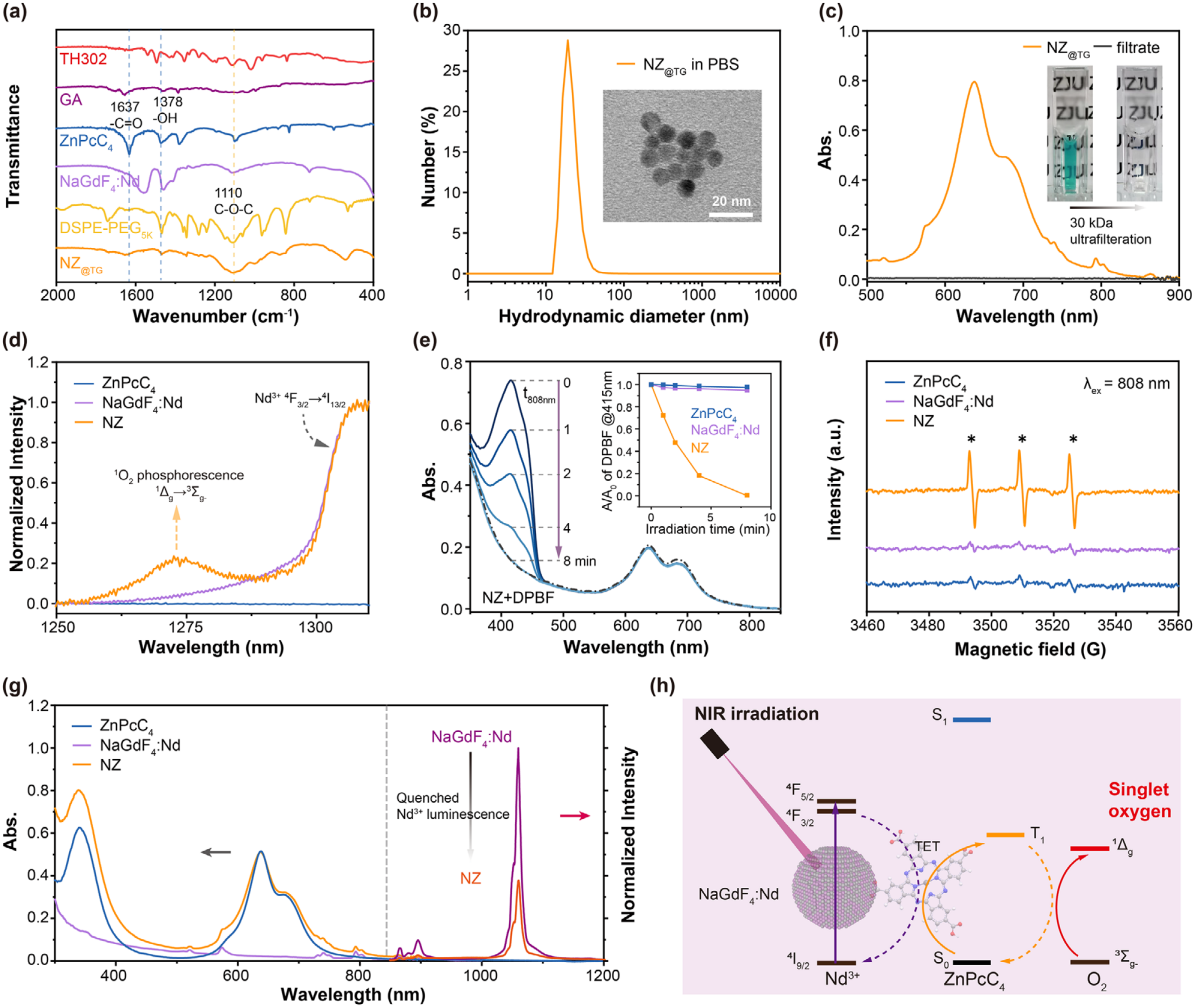
a) FTIR spectra of NZ_@TG_ and each component in the nanocomposites. b) Hydrodynamic diameter of NZ_@TG_ in PBS measured by DLS. (Insert: TEM image of NZ_@TG_.) c) Absorption spectra and digital photos of NZ_@TG_ before ultrafiltration and the filtrate of NZ_@TG_ after ultrafiltration. d) Singlet oxygen phosphorescence in NZ solution (CCl_4_) under 808 nm NIR irradiation. e) Absorption spectra showing DPBF degradation by singlet oxygen generated by NZ under 808 nm NIR irradiation. (Insert: DPBF oxidation over irradiation time by ZnPcC_4_, NaGdF_4_:Nd, and NZ.) f) Electron spin resonance (ESR) spectra showing singlet oxygen generation using TEMP as spin trapper. g) Absorption spectra of ZnPcC_4_, NaGdF_4_:Nd, and NZ, and 808 nm laser excited photoluminescence spectra of NaGdF_4_:Nd and NZ. h) Schematic illustration of the photophysical process of singlet oxygen generation mediated by NIR activatable lanthanide doped inorganic nanocrystal/organic dye conjugates.

The NZ_@TG_ nanoplatform was fabricated as illustrated in Scheme 1. ZnPcC_4_ was incorporated onto NaGdF_4_:Nd nanocrystals to form nanocrystal‐organic molecule nanocomposites (NZ) via carboxyl coordination (Figures S1–S6, Supporting Information). The hydrophobic nanocomposites were then transferred to aqueous media using an amphiphilic DSPE‐PEG5000 coating to enhance biocompatibility. During this process, the hypoxia‐activated prodrug TH302 and the mitochondria‐targeting ligand GA were encapsulated, forming the complete NZ_@TG_ nanoplatform (**Figure** **1**a; Figures S7–S12, Supporting Information). Variants containing only TH302 or lacking encapsulated agents were prepared with similar approaches, and termed as NZ_@T_ and NZ, respectively. Dynamic light scattering (DLS) measurements confirmed the uniform size distribution of NZ_@TG_, with an average hydrodynamic diameter of ≈21 nm in phosphate‐buffered saline (PBS) (Figure 1b). The structural integrity of the NaGdF_4_:Nd‐ZnPcC_4_ interface was demonstrated by the absence of detached ZnPcC_4_ in the ultrafiltration filtrate of the NZ_@TG_ colloidal suspension (Figure 1c).

Under 808 nm NIR irradiation, the NZ nanocomposites exhibited excellent singlet oxygen (^1^O_2_) generation, as evidenced by the determination of characteristic phosphorescence signal of ^1^O_2_ at ≈1270 nm.^[^ ^33^ ^]^ Neither the NaGdF_4_:Nd nanocrystals nor the ZnPcC_4_ molecules alone produced the detectable phosphorescence signal, suggesting the importance of nanocrystal‐organic interaction for the NIR‐light responsive ROS production (Figure 1d). This ROS generation was further corroborated using the absorption probe 1,3‐diphenylisobenzofuran (DPBF) and the spin trapper 2,2,6,6‐tetramethylpiperidine (TEMP).^[^ ^34^ ^]^ The decline in DPBF absorption at 415 nm confirmed the oxidation of DPBF by ROS, while the electron spin resonance (ESR) spectra revealed the formation of 2,2,6,6‐tetramethylpiperidinooxy (TEMPO), the adduct product of TEMP and ^1^O_2_ in NZ suspension upon NIR irradiation (Figure 1e,f; Figures S13–S16, Supporting Information). We reason that interfacial energy transfer between the nanocrystals and ZnPcC_4_ underpins the robust ^1^O_2_ generation. Nd^3+^‐doped nanocrystals exhibit an intense luminescence peak at ≈1060 nm and weaker emissions at ≈864 and ≈899 nm under 808 nm excitation, corresponding to radiative transitions from the ^4^F_3/2_ state (Figure 1g). Although the luminescence of Nd^3+^ does not overlap with the absorption spectrum of ZnPcC_4_, the energy of the lowest excited triplet state of ZnPcC_4_ (ΔE_S0‐T1_, ≈1.13 eV) aligns closely with the ^4^F_3/2_ excitation state of Nd^3+^ (≈1.43 eV),^[^ ^35^ ^]^ enabling triplet energy transfer (TET) from Nd^3+^ to ZnPcC_4_. This was confirmed by quenched luminescence and reduced luminescence lifetime of Nd^3+^ in the presence of ZnPcC_4_ (Figure 1g; Figure S17, Supporting Information). The energy transfer mechanism is illustrated in Figure 1h, where energy harvested by Nd^3+^ from 808 nm photons is transferred to ZnPcC_4_’s triplets, subsequently producing ^1^O_2_. This design successfully shifts the excitation wavelength of phthalocyanine from the red to the NIR region without the need of modifying its molecular structure. Notably, the NaGdF_4_:Nd‐ZnPcC_4_ hybrid system adopted short‐range energy transfer from excited Nd^3+^ lanthanide ions to molecular triplet states at the organic/inorganic interface for efficient near‐infrared photosensitization via direct lanthanide‐triplet sensitization.^[^ ^36^, ^37^, ^38^ ^]^ As a result, we can bypass major energy loss steps including upconversion and intersystem crossing (ISC), existing in the conventional upconversion routes adopted in most lanthanide‐based nanoparticle/organic photosensitizer composite systems.^[^ ^19^, ^39^ ^]^

### 2.2 In Vitro Therapeutic Effect under NIR Irradiation

**Figure 2:**
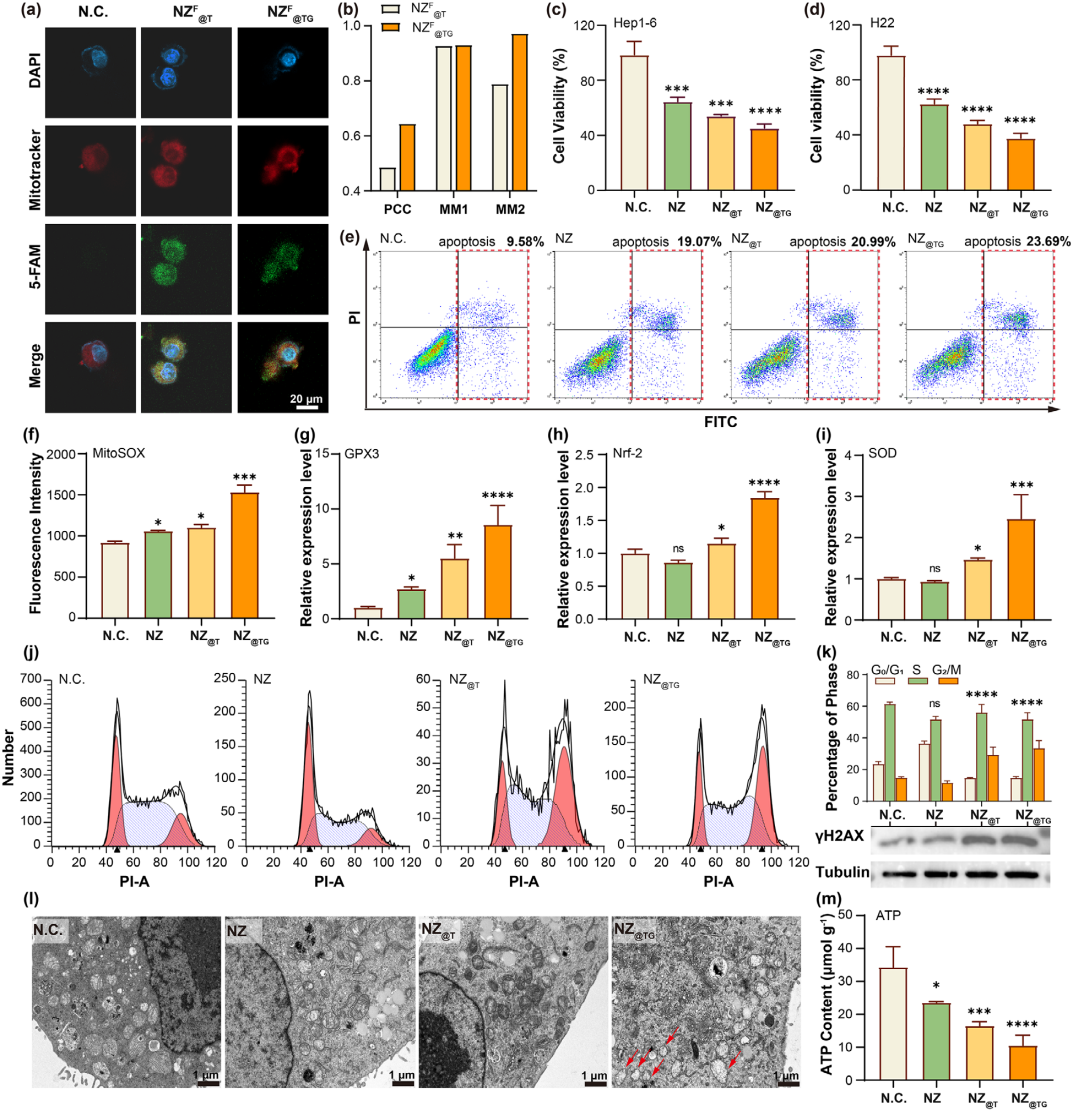
Phototherapy induced cell killing effects in vitro. a) Confocal images showing mitochondrial targeting effect of nanoparticles conjugated with GA. b) Quantification of co‐localization of nanocomposites and mitochondria with/without GA incorporation. Cell viability of c) Hep1‐6 and d) H22 cell lines under different treatment. e) Flow cytometry results of apoptosis of Hep1‐6 with different treatment strategies. f) Mitochondrial ROS level indicated by fluorescence intensity. Quantification of relative expression level of oxidative stress related genes via enzyme linked immunosorbent assay (ELISA): g) GPX3, h) Nrf‐2, and i) SOD. j) Identification of cell cycle arrest by flow cytometry. k) Quantification of phases in cell cycles and western blot identifying up‐regulation of γH2AX after treatment. l) Identification of mitochondrial damage via transmission electron microscope images. m) Quantification of intracellular ATP content. Values are expressed as mean ± SD. (vs N.C. group, ns: *p* > 0.05; *: *p* < 0.05; **: *p* < 0.01; ***: *p* < 0.001; ****: *p* < 0.0001 determined using one‐way ANOVA followed by Turkey's test).

To maximize PDT efficacy under NIR irradiation, NZ_@TG_ was designed to selectively target mitochondria. To confirm this ability, 5‐carboxyfluorescein (5‐FAM) was labelled to NZ_@TG_ to monitor the intracellular distribution of the nanocomposites (Figure S18, Supporting Information). After cell incubation, confocal imaging revealed strong colocalization between the green fluorescence of 5‐FAM and the red fluorescence of commercial mitotracker, indicating effective mitochondrial targeting of NZ_@TG_ in Hep1‐6 cells (**Figure** **2**a). Quantitative analysis further validated this observation, with higher Pearson's colocalization coefficient (PCC) and Mander's M2 (MM2) values for NZ_@TG_ compared to NZ_@T_ (lacking GA), demonstrating enhanced mitochondrial affinity due to the incorporation of GA with the nanocomposites (Figure 2b; and Table S1, Supporting Information). Additionally, the incorporation of TH302 and GA introduced minimal influence on ^1^O_2_ sensitization performance of the nanocomposites under NIR excitation (Figure S19, Supporting Information).

The therapeutic efficacy of NZ_@TG_ was then evaluated in vitro using the CCK‐8 assay on Hep1‐6 and H22 cancer cell lines. Under 808 nm NIR irradiation, NZ_@TG_ exhibited the highest cytotoxicity among the tested groups, significantly reducing cell viability while showing negligible dark cytotoxicity (Figure 2c,d; Figure S20, Supporting Information). The incorporation of both the mitochondria‐targeting ligand and the hypoxia‐activated prodrug in NZ_@TG_ contributed to its superior therapeutic performance compared to NZ and NZ_@T_ groups. Flow cytometry analysis confirmed that NZ_@TG_ induced the highest levels of apoptosis under NIR irradiation (Figure 2e; Figure S21, Supporting Information). This was accompanied by upregulation of apoptosis‐related proteins such as Caspase‐3 and BAX, and downregulation of the anti‐apoptotic protein such as Bcl‐2 (Figure S22, Supporting Information).

We attributed the enhancement in NIR‐activated cytotoxicity to localized ROS enrichment within the mitochondrial microenvironment. Using 2′,7′‐dichlorofluorescin diacetate (DCFH‐DA) as intracellular ROS indicator, a ≈73% increase in overall cellular ROS levels was observed when the PDT agent was targeted to mitochondria (Figure S23, Supporting Information). This ROS enrichment was particularly pronounced in mitochondrial regions, as shown by the fluorescence intensity of the mitochondrial ROS indicator, MitoSOX, which was ≈3.92 times higher in NZ_@TG_ treated cells than that in NZ‐treated groups (Figure 2f; Figure S24, Supporting Information).

ROS accumulation induced substantial oxidative damage, as evidenced by the upregulation of oxidative stress‐related proteins, including GPX3, Nrf‐2, and SOD (Figure 2g–i).^[^ ^40^ ^]^ The hypoxia generated during PDT facilitated the activation of TH302 prodrug to form toxic Br‐IPM, consequently leading to significant DNA damage.^[^ ^41^, ^42^, ^43^ ^]^ Without photodynamic activation, TH302 alone could not introduce substantial disturbance to cell viability or apoptosis even at high concentrations (Figure S25, Supporting Information). By monitoring the cells with various treatments, we observed the most serious cell cycle arrest at G_2_/M stage in the TH302 incorporated groups (Figure 2j,k). DNA impairment introduced by hypoxia activated TH302 was also confirmed by γH2AX upregulation as shown in Figure 2k. Such DNA impairment inhibits rapid cancer cell proliferation, which is a critical step in slowing tumor progression and creating a therapeutic window to enhance treatment efficacy.

Transmission electron microscope images further demonstrated that NZ_@TG_ caused severe mitochondrial damages under NIR irradiation, including mitochondrial swelling, disruption of ridges, decreased ridge density, and damage to the mitochondria‐endoplasmic reticulum interface accompanied by vesicle formation (Figure 2l). This structural disruption resulted in impaired mitochondrial functions, characterized by decreased ATP production and reduced mitochondrial membrane potential, as indicated by a lower JC‐1 monomer/polymer ratio (Figure 2m; Figure S26, Supporting Information). The localized oxidative damage also triggered positive feedback, amplifying ROS production and reinforcing oxidative stress. Thus, strategic enrichment of NIR photo‐generated ROS within mitochondrial regions could initiate a cascade of events, inducing severe oxidative stress that culminates in tumor cell apoptosis.

### 2.3 In Vitro Activation of Anti‐Tumor Immune Responses

**Figure 3:**
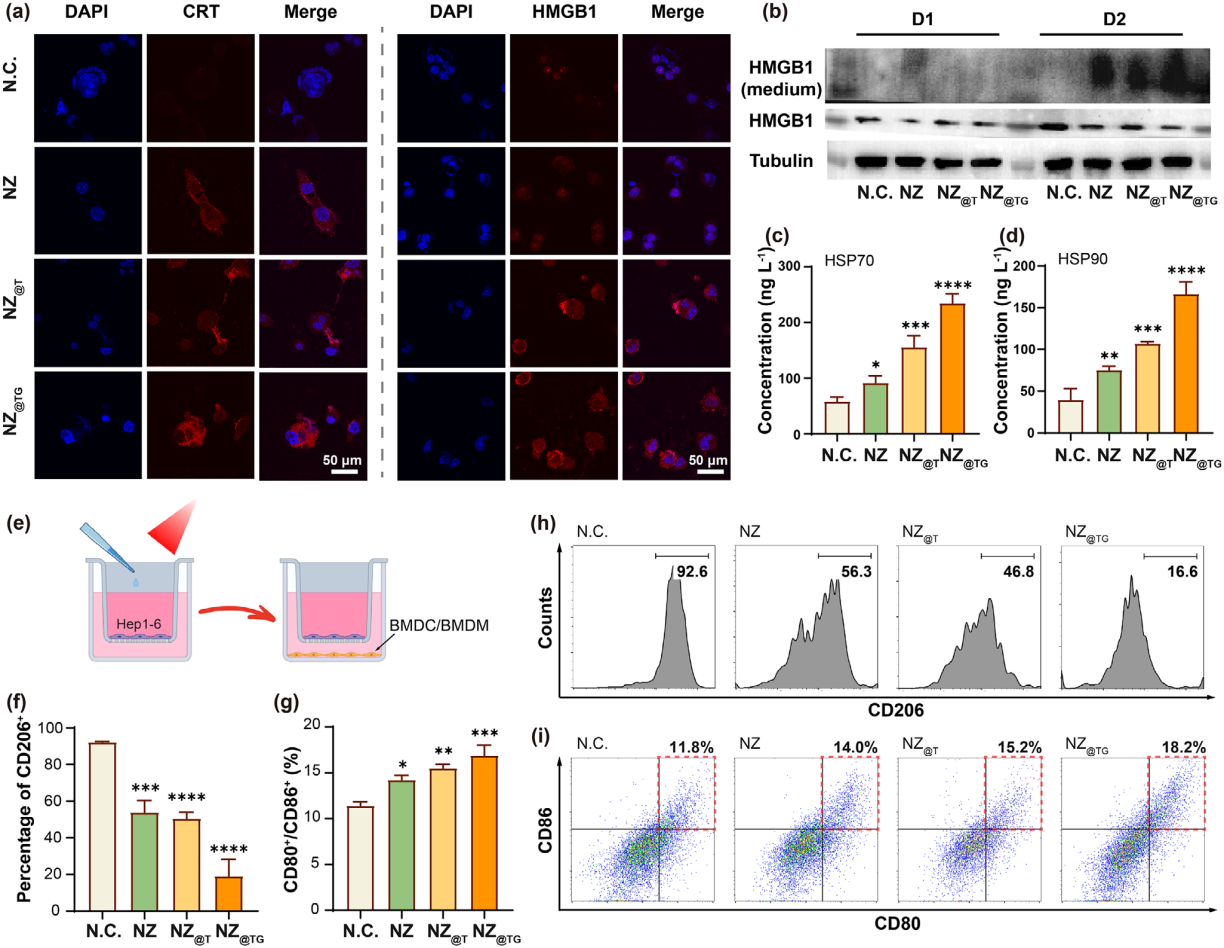
In vitro investigation on phototherapy‐initiated ICD. a) Identification of CRT translocation to plasma membranes and HMGB1 secretion by confocal laser scanning microscope (CLSM). b) Western blot showing HMGB1 secretion into medium. Quantification of c) HSP70 and d) HSP90 in cell supernatant by ELISA test (*n* = 3). e) Schematic illustration of in vitro induction of BMDC/BMDM cell differentiation by combined phototherapy of Hep1‐6 cells. Quantification of f) CD206^+^ cells and g) CD80^+^/CD86^+^ cells. h) Percentage of M2 phenotype after co‐culturing BMDM cells with Hep1‐6 accepting various treatment tested by immune cell flow cytometry. i) Immune cell flow cytometry results of BMDC cells co‐cultured with Hep1‐6 after various treatments. Values are expressed as mean ± SD. (vs N.C. group, ns: *p* > 0.05; *: *p* < 0.05; **: *p* < 0.01; ***: *p* < 0.001; ****: *p* < 0.0001 determined using one‐way ANOVA followed by Turkey's test).

Oxidative damage induced by PDT can promote ICD, triggering the release of DAMPs and activating anti‐tumor immune responses.^[^ ^44^ ^]^ To validate such an effect, key DAMPs, including calreticulin (CRT), high mobility group box 1 (HMGB1), and heat shock proteins (HSP70 and HSP90) were assessed in Hep1‐6 cells after various treatments. Among all treatment groups, NZ_@TG_ exhibited the most intense fluorescence signals of CRT and HMGB1 (**Figure** **3**a; Figure S27, Supporting Information). Western blot analysis further confirmed HMGB1 secretion into the extracellular medium 48 h after NIR treatment with NZ_@TG_ (Figure 3b). ELISA results demonstrated significantly elevated levels of HSP70 and HSP90 in the cell supernatants of the NZ_@TG_ group compared to other groups (Figure 3c,d). These results indicate the robust induction of ICD with NIR‐irradiated mitochondria‐targeting PDT.

To evaluate the impact of PDT‐induced DAMPs on immune cell activation, mouse‐derived primary bone marrow‐derived cells (BMDCs) and macrophages (BMDMs) were co‐incubated with NIR‐irradiated Hep1‐6 cells under various conditions (Figure 3e). Flow cytometry analysis revealed that the NZ_@TG_‐treated cells significantly suppressed the expression of CD206, a marker for immunosuppressive M2 macrophages, from 92.6% to 16.6% (Figure 3f,h).^[^ ^45^ ^]^ Concurrently, the expression of CD80 and CD86, markers of mature DCs, was markedly increased in BMDCs co‐cultured with NZ_@TG_‐treated cells (Figure 3g,i). These results suggest that NZ_@TG_‐induced ICD can effectively polarize macrophages toward the pro‐inflammatory M1 phenotype and promote dendritic cell maturation, both of which are critical for initiating systemic anti‐tumor immunity.

Taken together, the above findings highlight the ability of mitochondria‐targeting‐enhanced NIR PDT to not only cause localized cell death but also activate immune cells, setting the stage for long‐term systemic anti‐tumor responses.

### 2.4 In Vivo Therapeutic Effect and Mechanism Investigation

**Figure 4:**
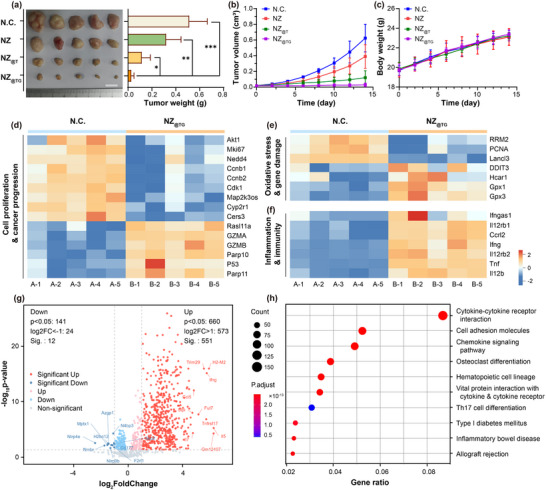
In vivo anti‐tumor efficacy and mechanisms of phototherapy on subcutaneous H22 liver tumor models. a) Digital images and weight of tumor tissues of various groups (*n* = 5). Variation of b) tumor volume and c) body weight in various groups (*n* = 5) bearing subcutaneous tumor over time. Heat map in pertinent to transcriptomics of subcutaneous H22 liver tumor organized by d) cell proliferation and cancer progression; e) oxidative stress and gene damage; f) inflammation and immunity. g) Volcano plot of expression variation in immune response relative genes. h) Bubble map based on KEGG channel analysis. Values are expressed as mean ± SD. (vs NZ_@TG_ group, ns: *p* > 0.05; *: *p* < 0.05; **: *p* < 0.01; ***: *p* < 0.001; ****: *p* < 0.0001 determined using one‐way ANOVA followed by Turkey's test).

The in vivo therapeutic potential of the NZ_@TG_ nanoplatform was first evaluated in subcutaneous H22 liver tumor‐bearing mice. Male BalB/C mice were obtained from Zhejiang University Laboratory Animal Center. H22 cell suspensions were subcutaneously injected to 7‐week‐old mice at right groin areas to construct subcutaneous tumor models. After 808 nm NIR irradiation (0.8 W, 0.4 cm^2^ spot size, 8 min), the NZ_@TG_ treated group exhibited significant enhanced tumor suppression compared to other control groups, as evidenced by reduced tumor weight and volume (**Figure** **4**a,b; Figure S28, Supporting Information). Importantly, the body weight of mice in all groups remained similar throughout the whole observation period, indicating the biosafety of the treatments (Figure 4c).

To gain insights into the underlying mechanisms, transcriptome sequencing was conducted on tumor tissues 14 days post‐treatment. Differential expression analysis revealed 4356 upregulated genes and 527 downregulated genes after the treatments (Figure S29, Supporting Information). As shown in the heat maps, downregulation of Nedd4, Map2k3os, Cyp2rl, and Cers3 and upregulation of the classical cancer suppression marker P53 reflected the inhibited tumor progression after the NZ_@TG_ treatment (Figure 4d; Figures S30 and S31, Supporting Information).^[^ ^46^, ^47^ ^]^ Variation in the pro‐apoptosis GZMA, GZMB, Parp10, and Parp11 corresponded to the amplified cell apoptosis in previous cellular investigation.^[^ ^48^, ^49^ ^]^ Particularly, we observed that expression of genes involved in the cell cycle for proliferation, such as Mki67, Ccnb1, Ccnb2, and Cdk1 were downregulated, suggesting cell cycle stasis in the highly proliferating cancer cells attributed to TH302 activation.^[^ ^50^, ^51^ ^]^ Moreover, elevated expression of Hcar1 and the Gpx family genes indicated the activation of oxidative stress response mechanisms following PDT, while upregulation of the damage‐associated gene DDIT3 and downregulation of repair‐related gene RRM2 and PCNA suggested the synergistic effects of PDT‐induced oxidative damage and TH302‐activated chemotherapy (Figure 4e).^[^ ^52^, ^53^, ^54^ ^]^

More importantly, the NZ_@TG_ treatment also activated pro‐inflammation and immune‐promoting pathways as evidenced by upregulated expression of inflammation/immunity‐related genes, such as Ifngas1 and Il12rb1 (Figure 4f). Overall expression of immunology associated mRNA was summarized in the volcano map in Figure 4g. As can be seen, the NZ_@TG_ treatment significantly increased H2‐M2 and Infg expression, which facilitated MHC‐I and INF‐γ upregulation contributing to immune response by inducing M1 macrophage polarization for tumor antigen presentation.^[^ ^55^ ^]^ Kyoto Encyclopedia of Genes and Genomes (KEGG) pathway enrichment analysis highlighted the close correlation between the overexpressed genes and cytokines and chemokines related immune responses pathways (Figure 4h).

The limited tissue penetration of visible light has long been regarded as a major limitation of conventional PDT, restricting its application to superficial tumors.^[^ ^56^, ^57^, ^58^ ^]^ In contrast, NIR light offers deeper tissue penetration, which makes it suitable for treating deeply seated malignancies.^[^ ^56^ ^]^ To further validate this potential, we applied the nanoplatform in treating orthotopic liver tumor models, where tumors were located beneath multiple tissue layers, including skin and muscle with a thickness of up to 1 cm. This depth exceeds the penetration limits of visible light, thus giving us confidence to explore the capability of our mitochondria‐targeting enhanced NIR PDT to eradicate tumors at deeper sites rather than superficial subcutaneous areas.

**Figure 5:**
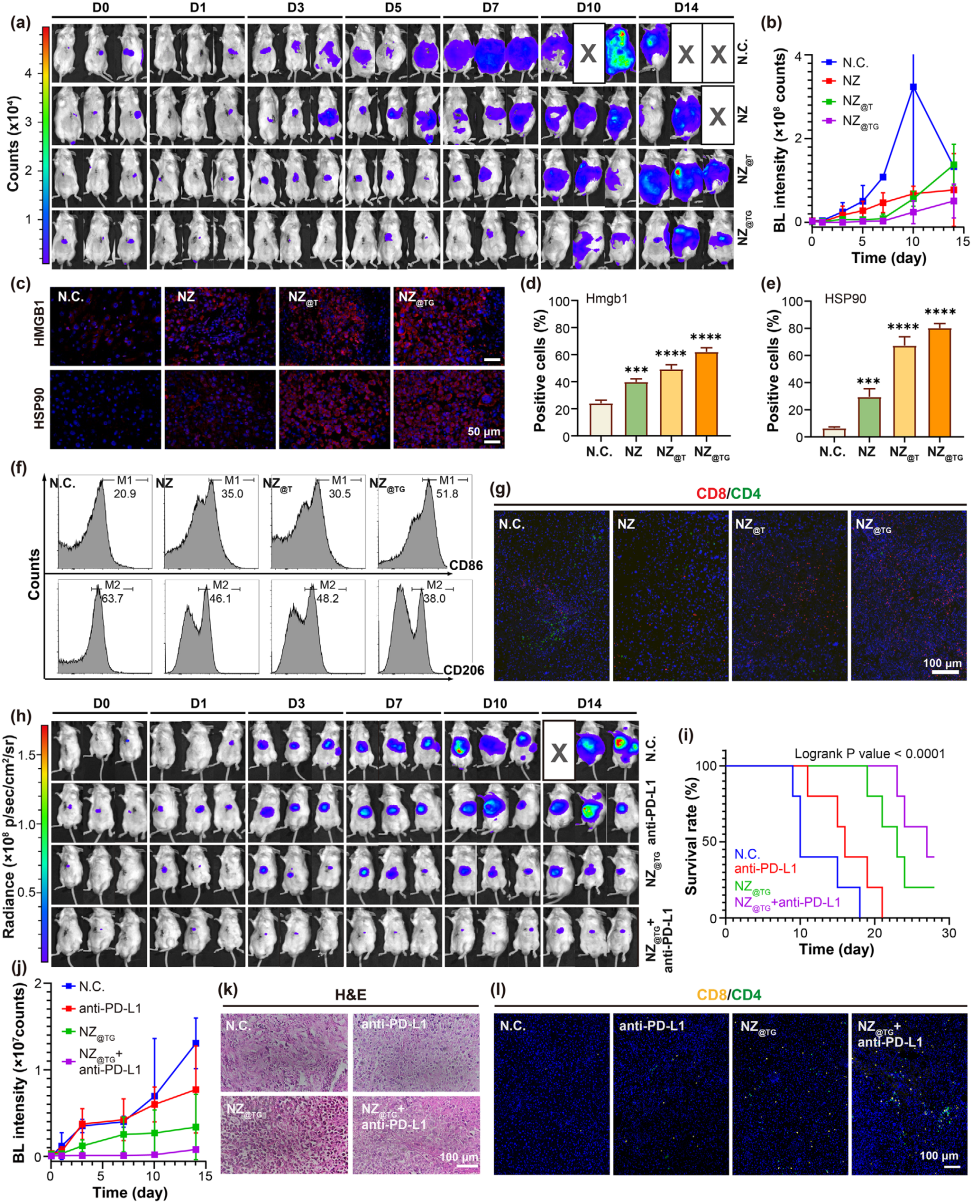
Deep tissue penetrating PDT activated anti‐tumor immunity for orthotopic H22 liver tumor treatment under NIR irradiation and combined immune checkpoint blockade therapy. a) BL images of orthotopic liver H22 tumor‐bearing mice from day 0 to day 14. b) Quantification of BL intensities over time in orthotopic tumor‐bearing mice (*n* = 3). c) Immunofluorescence images of HMGB1 and HSP90 stained tissue slices from orthotopic H22 liver tumors after various treatments. Quantification of d) HMGB1 and e) HSP90 positive cells in (c) (*n* = 3). f) Percentage of M1 and M2 phenotype in orthotopic H22 liver tumors with various treatments quantified by immune cell flow cytometry. g) CD8 and CD4 staining of the orthotopic liver H22 tumor tissues on day 14 post‐treatment. h) BL images of orthotopic liver H22 tumor‐bearing mice accepting combined therapy from day 0 to day 14 (anti‐PD‐L1 was administrated on day 1, 4, and 7). i) Survival curve of mice accepting combined therapy (*n* = 5). j) Quantification of BL intensities over time in orthotopic tumor‐bearing mice accepting combined therapy (*n* = 3). k) H&E staining of tumor tissues on day 14 post combined treatment. l) CD8 and CD4 staining of tumor tissues on day 14 post combined treatment. Values are expressed as mean ± SD. (vs N.C. group, ns: *p* > 0.05; *: *p* < 0.05; **: *p* < 0.01; ***: *p* < 0.001; ****: *p* < 0.0001 determined using one‐way ANOVA followed by Turkey's test). Kaplan‐Meier method followed by a log‐rank test was used for survival analysis.

The orthotopic liver tumor models were constructed by orthotopically injecting luciferase‐labelled H22 cell suspension to 7‐week‐old male BalB/C mice. One week later, various nanomaterials were intratumorally given to the mice 4 h before implementing 8‐min 808 nm laser irradiation at 2 W cm^−2^. Orthotopic tumor‐bearing mice treated with NZ_@TG_ exhibited minimal tumor progression even on day 7 post treatment, as evidenced by bioluminescence (BL) imaging, while control groups showed rapid tumor growth and metastasis throughout the abdominal cavity (**Figure** **5**a,b). Importantly, the NZ_@TG_‐treated group achieved a 100% survival rate 14 days post‐treatment, compared to 33.3% and 83.3% mortality in the negative control group on days 5 and 14, respectively (Figure S32, Supporting Information).

In the tumor tissue samples, the NZ_@TG_‐treated group exhibited the most pronounced lipid peroxidation (4‐HNE staining), tumor cell ablation (H&E staining), cell proliferation suppression (Ki‐67 staining), and apoptosis (TUNEL staining) (Figures S33–S35, Supporting Information). Besides, good biosafety of the treatment modality was validated, as no abnormal results arose during observation of the tissues of major organs from various treatment groups with H&E staining (Figure S36, Supporting Information), and the blood test for the mice in various groups (Figures S37 and S38, Supporting Information).

### 2.5 Anti‐Tumor Immunity Potentiated by Mitochondria‐Targeting Enhanced NIR PDT

The in vivo anti‐tumor immune memory of the NZ_@TG_ nanoplatform was last verified by examining tumor samples from orthotopic H22 liver tumors after the PDT treatments. Orthotopic liver tumors treated with NZ_@TG_ exhibited robust ICD, characterized by increased expression and secretion of DAMPs (Figure 5c–e; Figure S39, Supporting Information). The enhanced ICD response promoted macrophage polarization toward pro‐inflammatory M1 phenotype, leading to a ≈1.48‐fold increase in M1 macrophages and a ≈40% reduction in immunosuppressive M2 macrophages compared with the negative control (N.C.) group (Figure 5f; Figure S40, Supporting Information).^[^ ^57^ ^]^ The shift in macrophage polarization, coupled with an elevated CD8^⁺^/CD4^⁺^ T cell ratio observed in tumors, lymph nodes, and spleens, demonstrated the successful activation of systemic anti‐tumor immune responses (Figure 5g; Figure S41, Supporting Information).

To evaluate the long‐term immune memory elicited by NZ_@TG_, recurrence tumor models were further established. Subcutaneous Hep1‐6 tumors in mice were first treated with intratumor injections of NZ_@TG_ or PBS (negative control, N.C.) followed by 808 nm NIR irradiation (Figure S42a, Supporting Information). Fourteen days post‐treatment, Hep1‐6 cells were re‐injected at the opposite flank to simulate tumor recurrence. Remarkably, tumor growth at the recurrence site was undetectable in the NZ_@TG_‐treated group by day 7, and only negligible tumor volumes were observed by day 14, compared to rapid tumor progression in the control group (Figure S42b, Supporting Information). The results clearly suggest the ability of NZ_@TG_ to elicit durable anti‐tumor immunity.

As the most widely adopted immunotherapy in clinical practice, ICB therapy functions by neutralizing receptors and ligands on tumor cells that inhibit T cell activation, thereby reversing immune escape mechanisms.^[^ ^58^, ^59^ ^]^ Combining mitochondria‐targeting enhanced NIR PDT with ICB therapy has the potential to further amplify immune responses. We further demonstrated this synergy in orthotopic H22 liver tumor‐bearing mice, where NZ_@TG_‐mediated PDT was administered on Day 0 to prime the immune system, followed by anti‐PD‐L1 therapy on Days 1, 4, and 7 to suppress immune escape (Figure S43, Supporting Information). The combined therapy significantly improved survival rates and suppressed tumor progression more effectively (Figure 5h–j). Histological analysis of tumor tissues on Day 14 revealed extensive edema and tissue damage in the combined therapy group (Figure 5k), while immunofluorescence staining of CD8^+^ and CD4^+^ cells showed enhanced T cell infiltration after the combined therapy (Figure 5l), confirming the mutual reinforcement between PDT‐induced immune activation and ICB‐mediated immune suppression reversal. Hence, it is promising to adopt the mitochondria‐targeting enhanced NIR PDT as the immune activation step to maximize the efficacy of clinically approved ICB therapies.

## 3 Conclusion

Photodynamic therapy is a promising therapeutic modality that can reverse the immune‐suppressive tumor environment. Targeting PDT agents to mitochondria can boost ROS generation owing to localized distribution of intracellular oxygen to maximize the pro‐inflammatory PDT effect. ROS easily disrupts the structure and physiological functions of mitochondria, which serve as more than the redox center of cells. Consumption of intracellular molecular oxygen would further exaggerate tumor hypoxia to facilitate the activation of hypoxia‐activated chemo‐prodrugs to initiate cascade chemotherapy. Following such a conception, we developed the mitochondria‐targeting enhanced PDT nanoplatform NZ_@TG_ and confirmed the designed therapeutic strategy. The PDT core consisted of Nd^3+^ doped inorganic nanocrystals and an organic photosensitizer, endowing the nanoplatform efficient ROS generation capability under 808 nm NIR irradiation. Owing to the enhanced tissue penetration of NIR irradiation, we demonstrated great therapeutic efficacy in orthotopic liver tumor models.

Upon NIR irradiation, NZ_@TG_ set off intense oxidative damage adjacent to mitochondria and continuingly consumed intracellular oxygen. Subsequently, TH302 was activated to the toxic form by exaggerated hypoxia to introduce DNA impairment and cause cell cycle arrest. Collectively, oxidative damage, mitochondrial disruption, and DNA impairment caused ICD that released DAMPs to activate systematic anti‐tumor immune responses. Specifically, pro‐inflammatory M1 polarization of macrophages was promoted while anti‐inflammatory M2 polarization was suppressed. Subsequently, CD8^+^ T cells were activated by tumor associated antigens presented on mature DCs and M1 macrophages for immune eradication of cancer cells. The robust anti‐tumor immunity not only contributed to the ablation of primary tumor, but more importantly reinforced the body with long‐term anti‐tumor immune memory that prevented further metastasis and recurrence and sensitized ICB therapy, hence providing an effective strategy for immune activation in cancer treatment research. Lanthanide‐based nanomaterials also make for a vast toolbox in multi‐modal real‐time monitoring of therapeutic efficacy and disease progression. Further exploration could be conducted on integration of diagnosis and treatment based on the NIR activatable pro‐inflammatory PDT nanoplatform proposed in this study to realize the real‐time reporting of the status of the immune system while activating systemic anti‐tumor immunity.

## 4 Experimental Section

### Synthesis of Hexagonal‐Phase Nd^3+^ Doped NaGdF_4_ Nanocrystals

The nanocrystals were synthesized via a modified thermal decomposition method.^[^ ^60^ ^]^ Solid Ln(CH_3_CO_2_)_3_ (Ln = Gd or Nd, 5 mmol) was mixed with oleic acid (20 mL) and 1‐octadecene (20 mL) in a 100‐mL three‐neck flask, followed by heating to 150 °C with N_2_ purging for 2 h to remove water and form lanthanide oleate complexes. Thereafter, the reactant was degassed for 30 min while cooling down to 110 °C. NaHF_2_ (10 mmol) powder was added into the reactant under N_2_ protection followed by 15 min degassing. The mixture was then heated to 310 °C for 40 min under N_2_ protection. The as‐prepared oleate‐capped NaGdF_4_:Nd nanocrystals were precipitated by ethanol and collected by centrifugation at 4,000 rpm for 8 min. The precipitates were re‐dispersed in cyclohexane and washed with ethanol three times, and finally re‐dispersed in cyclohexane (10 mL) for further modification.

### Preparation of NaGdF_4_:Nd‐ZnPcC_4_ Nanoconjugates

The organic/inorganic nanoconjugates were prepared via a ligand exchange method. Typically, oleate‐capped NaGdF_4_:Nd nanocrystals (50 mg) in cyclohexane were precipitated by ethanol and re‐dispersed in tetrahydrofuran (5 mL), which was then mixed with 1 ml methanol solution of ZnPcC_4_ (0.5 mg ml^−1^) in a 25 ml single neck flask. The mixture was stirred overnight with N_2_ protection. The nanoconjugates were collected by centrifugation at 20,000 rpm for 15 min and re‐dispersed in 2 ml of chloroform for further use.

### Hydrophilic Modification for NaGdF_4_:Nd‐ZnPcC_4_ Nanoconjugates

NaGdF_4_:Nd‐ZnPcC_4_ nanoconjugates (400 uL) was added dropwise into chloroform (3.6 mL) containing DSPE‐PEG5000 (40 mg) and stirred for 1 h under N_2_ protection, followed by heating at 50 °C until chloroform was fully evaporated. The sediment was dispersed in 2 ml phosphate buffer solution and purified by ultrafiltration. The collected hydrophilic nanocomposites were noted as NaGdF_4_:Nd‐ZnPcC_4_@DSPE‐PEG (NZ). Hydrophilic NaGdF_4_:Nd‐ZnPcC_4_@TH302/DSPE‐PEG (NZ_@T_) and NaGdF_4_:Nd‐ZnPcC_4_@TH302/GA/DSPE‐PEG (NZ_@TG_) were fabricated in the same manner except that a solution containing TH302 or TH302 and GA (1wt% of the nanocrystals) was added into the mixture together with NaGdF_4_:Nd‐ZnPcC_4_ nanoconjugates.

### Verification of NIR Irradiated Singlet Oxygen Generation in Solution

Nanocomposites (0.5 mg ml^−1^) consisting of NaGdF_4_:Nd and ZnPcC_4_ were dispersed in THF or PBS with 62.5 µM DPBF. The mixtures were then irradiated by 808 nm laser at 0.9 W cm^−2^ for various durations. Decrease in absorbance of DPBF at ≈415 nm reflects the generation of singlet oxygen. 5 mg ml^−1^ nanocomposites were dispersed in PBS with 100 mM TEMP as the spin trapper for singlet oxygen. The mixtures were irradiated by 808 nm laser at 2 W cm^−2^ for 10 min before injected into quartz capillary tubes for EPR tests.

### Cell Culture

Murine hepatoma Hep1‐6 and H22 cell lines were purchased from American Type Culture Collection, ATCC. Bone marrow‐derived macrophages (BMDMs) and bone marrow‐derived dendritic cells (BMDCs) were obtained through stimulation of colony stimulating factor and interleukin on extracted mouse bone marrow primary cells. All cells were cultured in a 5% CO_2_ cell incubator at 37 °C. Hep1‐6 cells were cultured with DMEM medium containing 10% v/v fetal bovine serum (FBS). H22 cells were cultured with 1640 medium containing 10% v/v FBS. BMDMs were cultured with DMEM medium containing 10% v/v FBS and 20 ng mL^−1^ macrophage‐stimulating factor. BMDCs were cultured with 1640 medium containing 10% v/v FBS, 10 mg mL^−1^ interleukin‐14 and 25 ng mL^−1^ granulocyte‐macrophage colony‐stimulating factor.

### In Vitro Cytotoxicity Investigation

Mitochondria targeting mediated by GA incorporation into the nanocomposites was verified by CLSM. Hep1‐6 cells were cultured with 5‐FAM‐labelled nanocomposites for 6 h before imaging. 5‐FAM shows a green emission upon 488 nm excitation. Mitochondria were labelled with mitotracker that exhibits red florescence when excited by 647 nm. In vitro cytotoxicity was measured by Cell Counting Kit (CCK‐8). Cells were seeded into 96‐well cell culture plates at 1 × 10^5^ per mL (for Hep1‐6 cells) or 2 × 10^5^ per mL (H22 cells). The final concentration of nanocomposites was kept at 200 µg mL^−1^. Control groups were treated with pure PBS. After 4–6 h of culturing in cell culture box for cellular intake of nanocomposites, all groups were irradiated with an 808 nm laser for 8 min (1 W cm^−2^, 0.4 cm^2^). Twenty‐four hours after phototherapy, 10 µL CCK‐8 reagent was added into each well, followed by 1 h of incubation before measuring OD at 450 nm.

### Cell Apoptosis

Annexin V‐FTIC/PI kit was used for measuring cell apoptosis. Cells were seeded into 6‐well plates before accepting various phototreatments. 24 h after phototherapy, cells were collected in 15 mL tubes and centrifuged for 5 min at 800 rpm. Sediments were redispersed in PBS and centrifuged again to obtain the final cell solution, which was mixed with PI (10 µL), Annexin V‐FTIC (5 µL), and binding buffer (500 µL), and incubated for 10 min at room temperature before tested by flow cytometry.

### Intracellular ROS Detection

DCFH‐DA was selected as the fluorescent probe for intracellular ROS. The obtained cell solutions from each group were mixed with probe solution and incubated for 20 min in the dark before tested by flow cytometry. MitoSOX RED was used as the fluorescent probe for mitochondrial singlet oxygen. The probe solution was diluted to 5 µm and preheated to 37 °C before adding to cells and incubating for 30 min. The cells were centrifuged at 800 rpm for 5 min and washed with PBS twice. The collected cell solution was filtered with 300 mesh filter before analyzed by cell flow cytometry.

### Cell Cycle Assessment

Cell cycle was tested by cell cycle detection assay. The cells from each group were re‐dispersed in 1 mL DNA staining solution with 10 µL permeabilization solution, which were then vortexed for 5–10 s. The cells were incubated for 30 min in the dark at room temperature before analyzed by flow cytometry.

### Mitochondria Damage Detection

Mitochondrial membrane integrity was investigated by JC‐1 detection assay from Beyotime. The obtained cell sediments were redispersed in 1 mL culture medium, where 1 mL of JC‐1 staining solution was added. The cells were cultured for 20 min at 37 °C, followed by centrifugation and washing. The final samples were filtered with 300 mesh filter before analyzed by cell flow cytometry. Cellular ATP detection assay was used to determine intracellular ATP content. The cells from each group were washed with PBS for three times and disrupted for 5 min on ice with 200 µL cell lysis buffer. Supernatants were collected after centrifugation, and added to ATP detection working solutions to obtain RLU values on chemiluminescence apparatus. ATP concentration was calculated comparing to the standard curve. Protein contents of each sample were determined by BCA method, which lead to the final intracellular ATP contents.

### Western Blotting

Cell lysates were extracted by cell lysis buffer (RIPA), dissolved for 30 min on ice and centrifuged at 12000×g for 30 min at 4 °C. The concentration of protein samples was detected by BCA kit (Thermo Fisher Scientific, MA, USA). Protein samples were subjected to sodium dodecyl sulfate (SDS)‐PAGE gels, followed by polyacrylamide gel electrophoresis. Electrotransfer was then performed to transfer the proteins into PVDF membrane (Bio‐Rad, CA, USA). After blocking with 5% skim milk for 1 h, the membrane was washed 3 times for 10 min each with 1× Tween Tris Buffered Saline (TBST). After 3 times of TBST washing, the membranes were incubated with the primary antibody overnight at 4 °C. Then the membrane was washed 3 times for 10 min each with 1× TBST, The washed membranes were incubated with the corresponding secondary antibody for 1–2 h at RT. After 3 times of TBST washing, the membranes were imaged by using Clarity Western ECL substrate (Bio‐Rad) and the signal was obtained by Bio‐red ChemiDoc Touch Imaging System.

### ELISA Test

Enzyme linked immunoscrbent assay (ELISA) was used to determine expression levels of various genes in tumor cells. Standard curve was established based on the OD at 450 nm of standard samples with various concentrations. Samples were incubated with dilution buffer, substrates and washed. The mixtures containing samples were blended with color developing agents A and B and the reaction was quenched before measurement.

### In Vivo Anti‐Tumor Efficacy Investigation

All animal experiments were performed following the protocols approved by the Ethics Committee of Sir Run Run Shaw Hospital, School of Medicine, Zhejiang University (Certificate No.: ZJU20230500). Male BalB/C mice were obtained from and approved by Zhejiang University Laboratory Animal Center (Certificate No.: ZJU20230500). To obtain subcutaneous H22 liver tumor model, 50 µL H22 cell suspension (1 × 10^7^ cells mL^−1^) labelled with luciferase was subcutaneously injected to 7‐week‐old mice with 1 week adaptive feeding at right groin area. One week after the construction of subcutaneous tumor, mice were anesthetized with pentobarbital to measure tumor size with vernier caliper. Tumor volume was calculated as follows:

(1) $$V=\frac{4}{3}\;\times \pi \times {\left(\frac{a+b}{2}+0.5\right)}^{3}\times \frac{1}{3}$$

where a and b are length and width of tumor in cm. 25 µg mL^−1^ (containing 5 µg mL^−1^ inorganic component) nanomaterials were injected into the tumor. Four hours later, mice were anesthetized with isoflurane while irradiated by 808 nm laser (2 W cm^−2^, 0.4 cm^2^) for 8 min.

To obtain orthotopic H22 liver tumor model, 10 µL H22 cell suspension (5 × 10^7^ cells mL^−1^) labelled with luciferase was orthotopically injected to 7‐week‐old mice with 1 week adaptive feeding at liver. One week later, mice were anesthetized with pentobarbital again and 25 µg mL^−1^ (containing 5 µg mL^−1^ inorganic component) nanomaterials were injected into the tumor. Four hours later, mice were anesthetized with isoflurane while irradiated by 808 nm laser (2 W cm^−2^, 0.4 cm^2^) for 8 min. In vivo small animal imaging was carried out after NIR irradiation. Orthotopic tumor‐bearing mice were weighed before anesthetization with pentobarbital. Each was injected with 150 µL solution of fluorescein sodium salt. Fifteen minutes later, the mice were placed into small animal imaging device and imaged with fixed exposure of 15 s.

At animal euthanization, blood of mice was collected for blood routine and blood biochemical analysis. Major organs of heart, spleen, liver, and kidney and tumor tissues were harvested from mice in each group and made into ≈5 µm sections. Sample sections were stained with H&E, Ki67, TUNEL, 4‐HNE, and corresponding fluorescent antibodies for immunofluorescence test.

### Statistical Analysis

Experiments in this study were performed using 3–5 samples or repeats for each experiment/group/condition. Representative staining images were presented. Results were expressed as Mean ± SD from at least three independent experiments. Statistical analysis was conducted with GraphPad Prism 9.0 software. Differences between two groups were analyzed using Student's *t* test, whereas one‐way ANOVA followed by the Turkey's test were adopted to examine the differences among three or more groups. Kaplan‐Meier method followed by a log rank test was used for survival analysis. *p* values < 0.05 were considered statistically significant. “*”, “**”, “***”, “****”, and “ns” represent *p* < 0.05, *p* < 0.01, *p* < 0.001, *p* < 0.0001, and *p* > 0.05, respectively.

## Conflict of Interest

The authors declare no conflict of interest.

## Supporting information



Supporting Information

## Data Availability

The data that support the findings of this study are available from the corresponding author upon reasonable request.
