# Supplementary material for: Amplifying Anti‐Tumor Immune Responses via Mitochondria‐Targeting Near‐Infrared Photodynamic Therapy
Source: Adv Sci (Weinh). 2025 Jun 9;12(33):e05525. doi: 10.1002/advs.202505525 (PMC12412487; doi:10.1002/advs.202505525)
Supplement: Supplementary file 1 — Supporting Information [file ADVS-12-e05525-s001.pdf]

ADVANCED SCIENCE
Open Access

Supporting Information

for *Adv. Sci.*, DOI 10.1002/advs.202505525

Amplifying Anti-Tumor Immune Responses via Mitochondria-Targeting Near-Infrared Photodynamic Therapy

Cheng-Ao Li, Junjie Nan, Qingxuan Ye, Bingzhu Zheng, Xiaomeng Dai, Jingya Li, Feng Wang, Huimin Ma, Yu Cheng, Jian Ruan, Weijia Fang, Peng Zhao, Renren Deng and Dong Cen**

Supporting Information

Amplifying Anti-Tumor Immune Responses via Mitochondria-Targeting Near-Infrared Photodynamic Therapy

Cheng-Ao Li, Junjie Nan, Qingxuan Ye, Bingzhu Zheng, Xiaomeng Dai, Jingya Li, Feng Wang, Huimin Ma, Yu Cheng, Jian Ruan, Weijia Fang, Peng Zhao, Renren Deng, and Dong Cen**

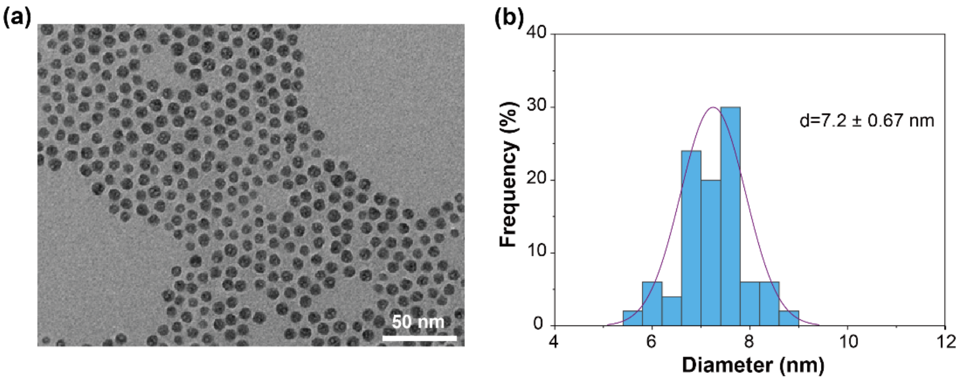

Figure S1. TEM image (**a**) and size distribution (**b**) of $NaGdF_4$:Nd nanocrystals as synthesized based on TEM characterization. The nanocrystals showed an average diameter of 7.2 nm with a narrow distribution.

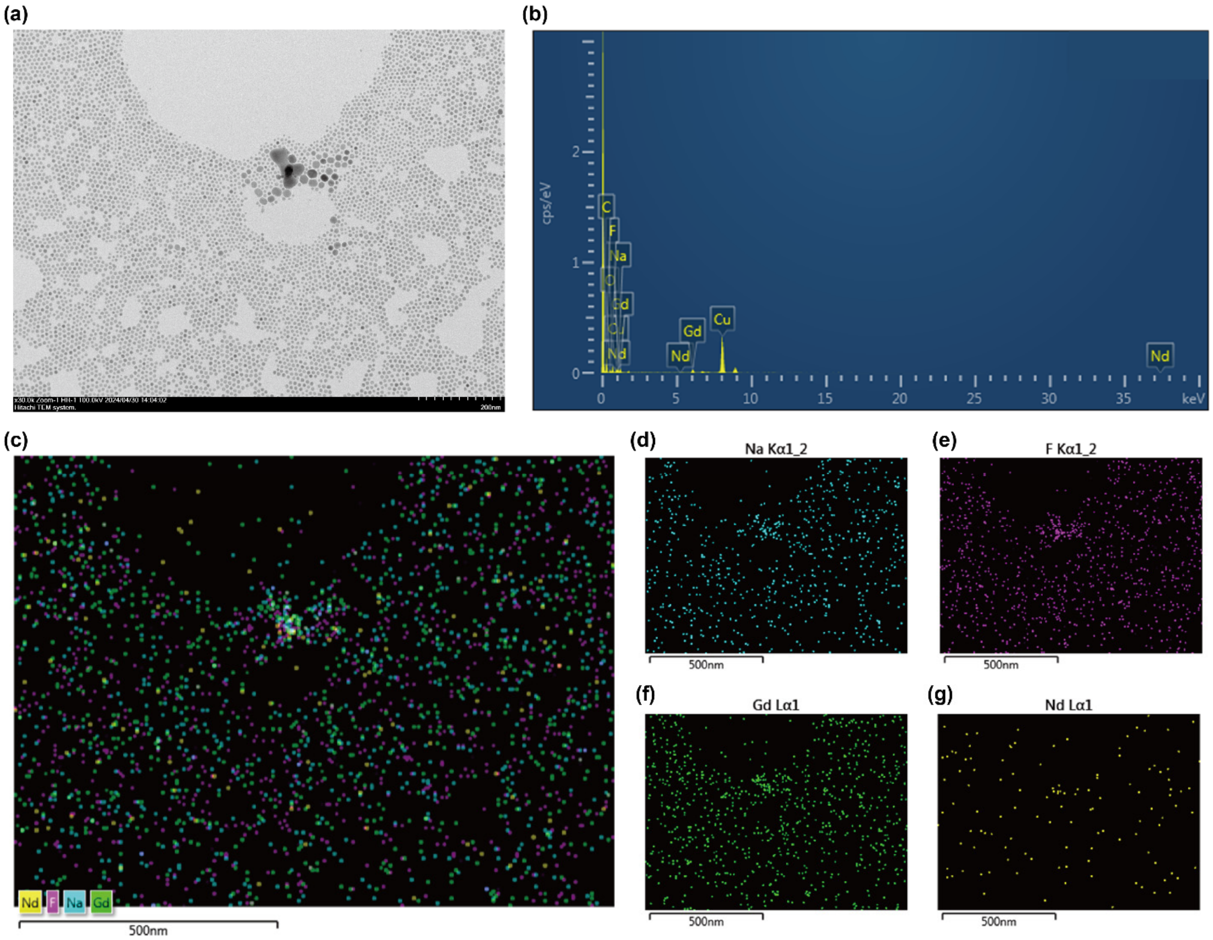

Figure S2. EDS mapping of $NaGdF_4$:Nd nanocrystals.
Nd^{3+} dopants dispersed uniformly in the $NaGdF_4$ matrix.

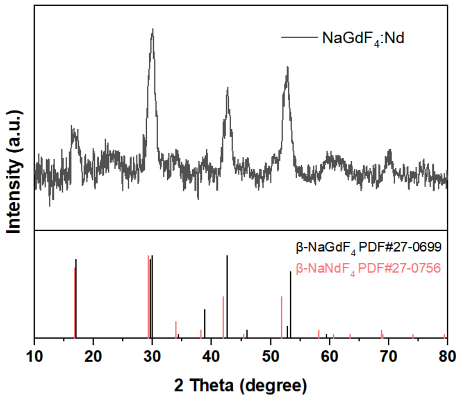

Figure S3 X-ray diffusion (XRD) pattern of NaGdF$_4$:Nd nanocrystals.
The nanocrystals exhibited a hexagonal (β) phase.

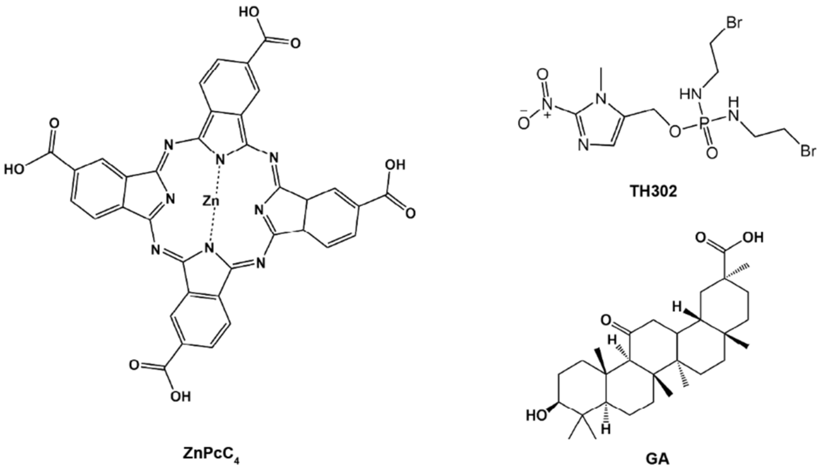

Figure S4 Molecular structures of ZnPcC$_4$, TH302, and GA.

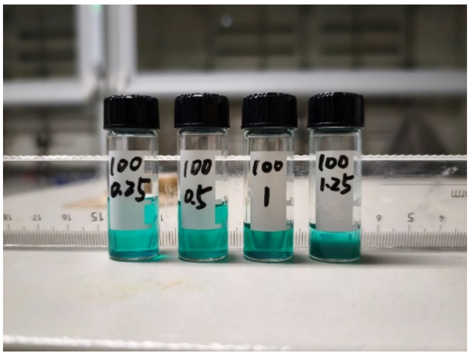

Figure S5 Digital photo of NaGdF$_4$:Nd-ZnPcC$_4$ in THF with various weight ratio of nanocrystals and photosensitizers (from left to right: 100:0.25, 100:0.5, 100:1, 100:1.25).

When the ratio reached 100:1.25, the nanocomposites began to aggregate and sediment to the bottom.

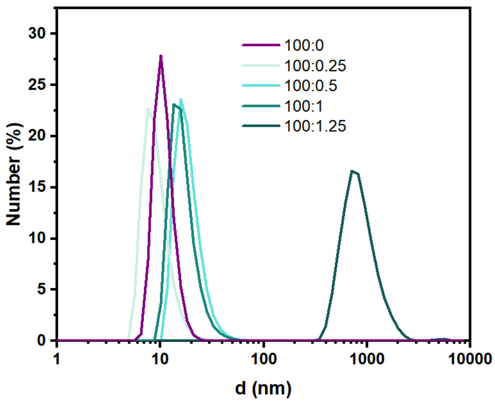

Figure S6 Dynamic light scattering (DLS) measured size distribution of $NaGdF_4$:Nd-ZnPcC4 nanocomposites in THF. The 100:0 sample refers to $NaGdF_4$:Nd nanocrystals as prepared.

Consistent with the digital photo, when the ratio reached 100:1.25, the nanocomposites seriously aggregated resulting in an average diameter of ~1,000 nm.

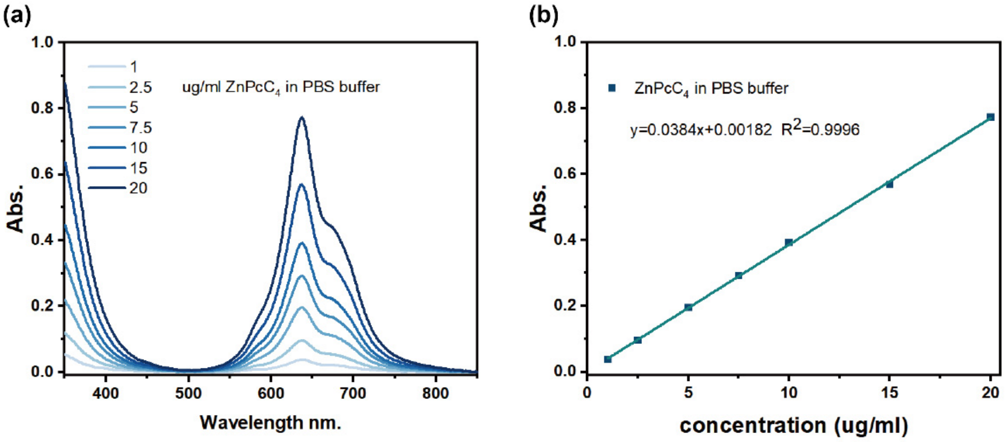

Figure S7 (**a**) Absorption spectra of $ZnPcC_4$ in PBS with various concentrations and (**b**) the extracted calibration curve of $ZnPcC_4$ in PBS based on absorption at 635.5 nm.

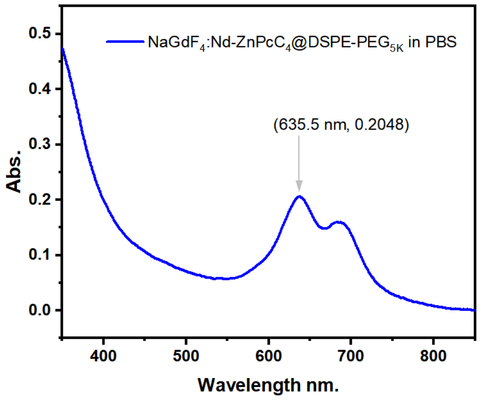

Figure S8 Absorption spectrum of $NaGdF_4$:Nd-$ZnPcC_4$@DSPE-PEG5000 in PBS containing 0.5 mg ml^{-1} nanocrystals.

The experiment setup was kept the same in Fig. S5. According to the absorption at 635.5 nm, a weight concentration of $ZnPcC_4$ of 5.28 μg ml^{-1} can be calculated, making a weight ratio of 94:1 of nanocrystal:photosensitizer, which is slightly lower than the 100:1 feed ratio for ligand exchange. Considering that ligand exchange is accompanied by release of surface capped oleic acid ligands, we deduce that the loading rate was close to 100%. Further calculation combined with nanocrystal size and cell size leads to an average conjugation of 9.36 $ZnPcC_4$ molecules on one nanocrystal.

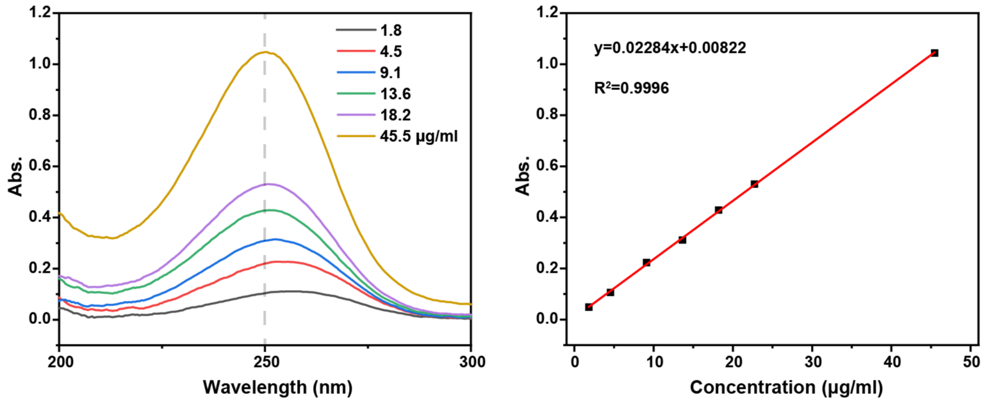

Figure S9 Absorption spectra of GA in PBS with various concentrations and the extracted calibration curve of GA in PBS based on absorption at 250 nm.

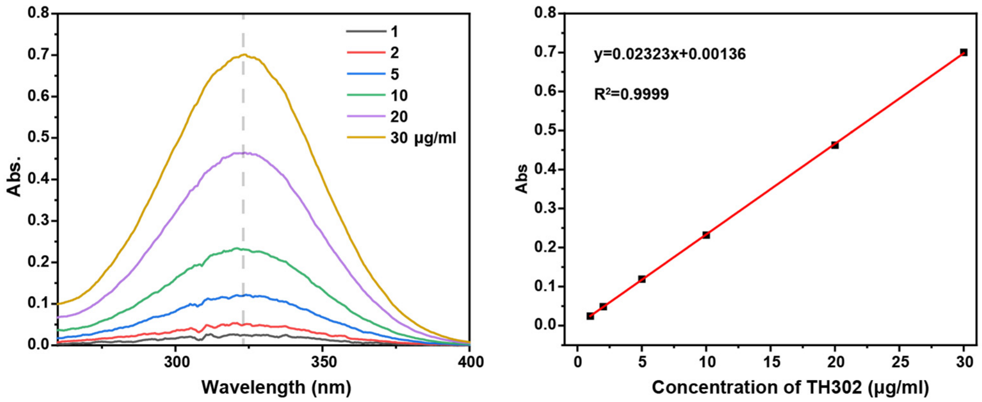

Figure S10 Absorption spectra of TH302 in PBS with various concentrations and the extracted calibration curve of TH302 in PBS based on absorption at 322 nm.

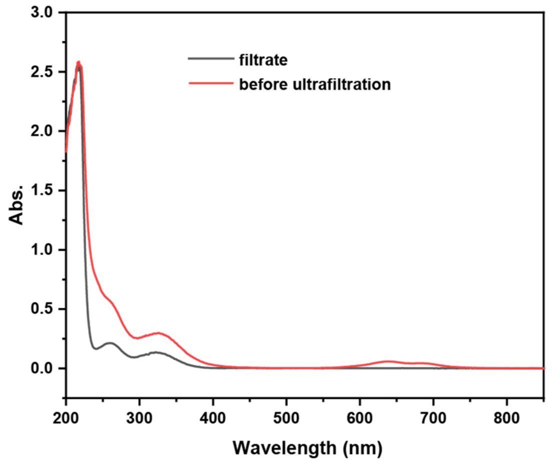

Figure S11 Absorption spectrum of NZ@TG in PBS containing 1 mg ml^{-1} nanocrystals.

According to the absorption at 250 nm and 322 nm before and after ultrafiltration, a weight concentration of GA of 8.23 μg ml^{-1} and a weight concentration of TH302 of 5.78 μg ml^{-1} in the filtrate can be calculated, making an incorporation rate of 17.7% and 42.2%, respectively (initial feed concentration was 10 μg mL^{-1} for both molecules).

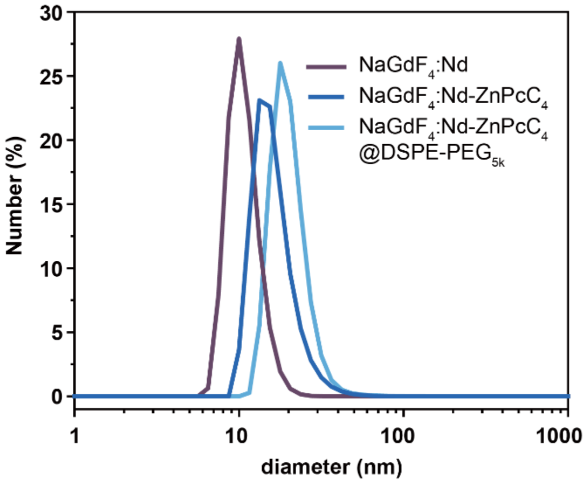

Figure S12 Size distribution of $NaGdF_4$:Nd in THF, $NaGdF_4$:Nd-$ZnPcC_4$ in THF, and $NaGdF_4$:Nd-$ZnPcC_4$@DSPE-PEG5000 in PBS measured by DLS.

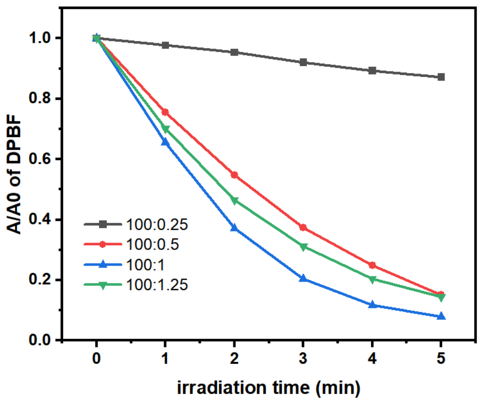

Figure S13 Degradation of DPBF by $^1O_2$ oxidation in THF under 808 nm irradiation at a power density of 0.9 W cm^{-2}.

NaGdF$_4$:Nd-ZnPcC$_4$ with various weight ratios was kept at 0.5 mg ml^{-1}. Doping concentration of Nd^{3+} was 2%. 100:1 sample exhibited the best $^1O_2$ generation ability.

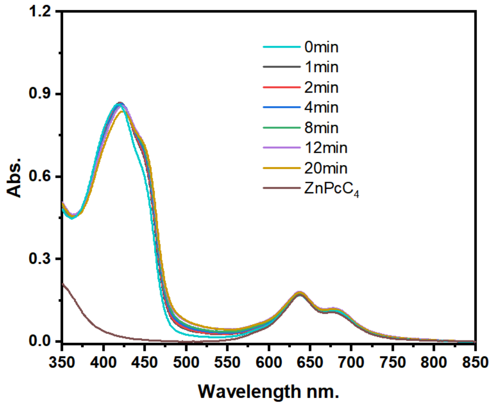

Figure S14 Absorption spectra of DPBF+ZnPcC$_4$ in PBS under 808 nm irradiation at a power density of 0.9 W cm^{-2}.

Absorption at 415 nm remained almost unchanged even after 20 min irradiation, verifying that ZnPcC$_4$ can not respond to 808 nm photons to generate $^1O_2$ and Nd-doped nanocrystals as an energy mediator is a must.

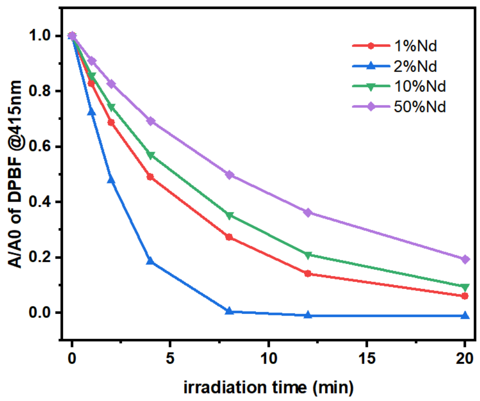

Figure S15 Degradation of DPBF by $^1O_2$ oxidation in THF under 808 nm irradiation at a power density of 0.9 W cm^{-2}.

$NaGdF_4$:Nd-$ZnPcC_4$ (100:1) with various Nd^{3+} doping concentrations was kept at 0.5 mg ml^{-1}. 2% sample exhibited the best $^1O_2$ generation ability.

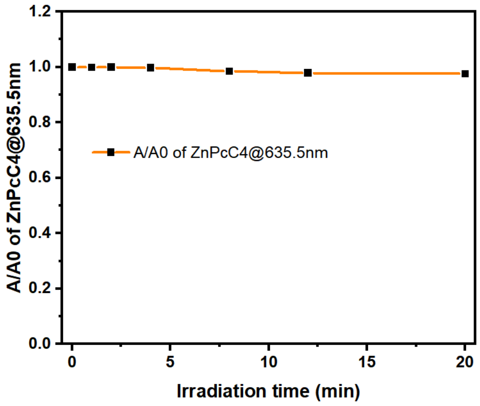

Figure S16 Normalized absorption of $NaGdF_4$:Nd-$ZnPcC_4$@DSPE-PEG5000 at 635.5 nm with 808 nm irradiation at 0.9 W cm^{-2}.

The absorption remained constant, validating great photo-stability of the photosensitizer in the nanocomposites upon NIR irradiation.

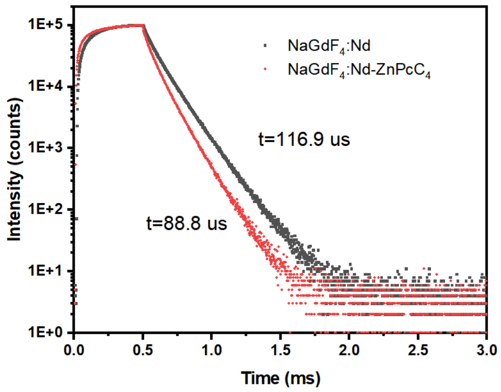

Figure S17 PL decay at 864 nm emission of Nd^{3+} upon 808 nm excitation.

Nd^{3+} PL lifetime dropped with $ZnPcC_4$ conjugation, demonstrating energy transfer from Nd^{3+} to $ZnPcC_4$.

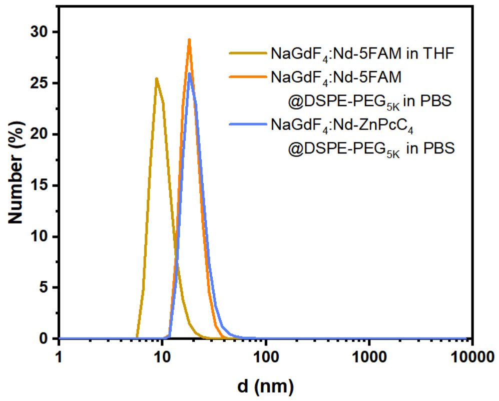

Figure S18 Size distribution of 5-FAM conjugated nanomaterials measured by DLS.

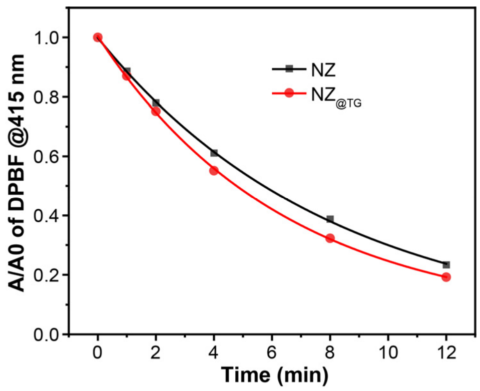

Figure S19 ROS generation under 808 nm irradiation via NZ and $NZ_{@TG}$ (0.5 mg mL^{-1}) detected by absorbance of DPBF at 415 nm.

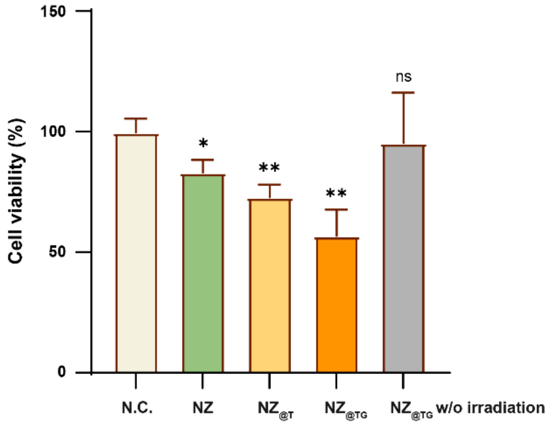

Figure S20 CCK-8 results of Hep1-6 cells with various treatments. Concentrations of nanomaterials were kept at 200 μg mL^{-1} in media.

The cells cultured with NZ@TG material in the dark showed minimal cell death, proving that the dark cytotoxicity of the final material is neglectable at this concentration.

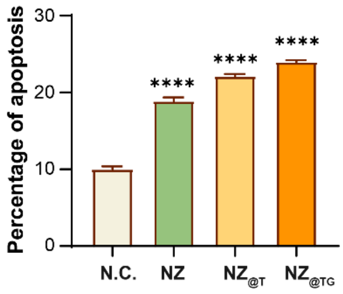

Figure S21 Quantification of apoptosis rate Hep1-6 cells with various treatments.

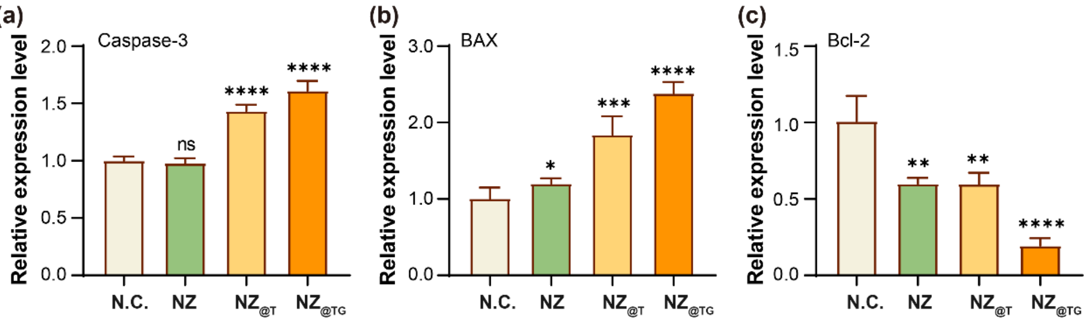

Figure S22 Quantification of relative expression level of apoptosis related proteins via ELISA: (**a**) Caspase-3, (**b**) BAX, and (**c**) Bcl-2.

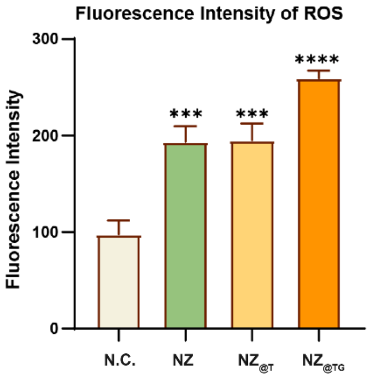

Figure S23 Intracellular ROS level demonstrated by fluorescence intensity of ROS probe DCFH-DA via cell flow cytometry.

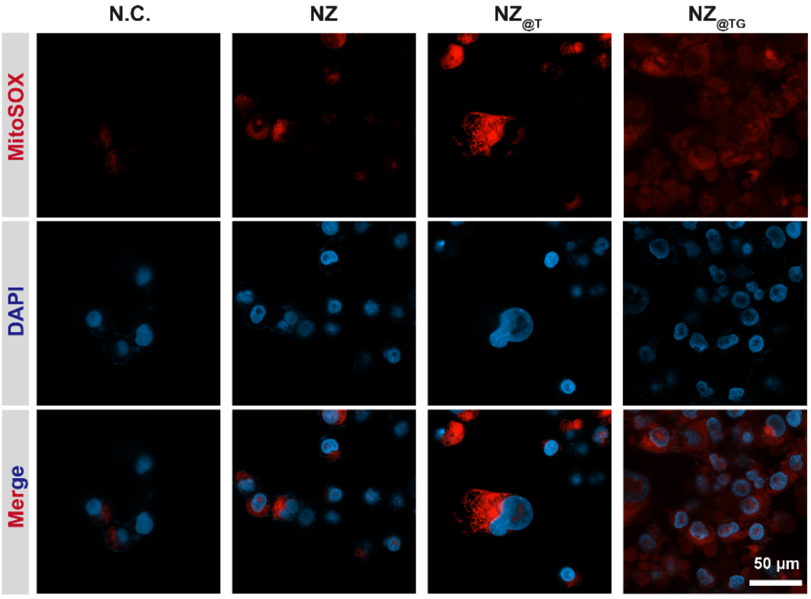

Figure S24 Identification of mitochondrial ROS by CLSM.

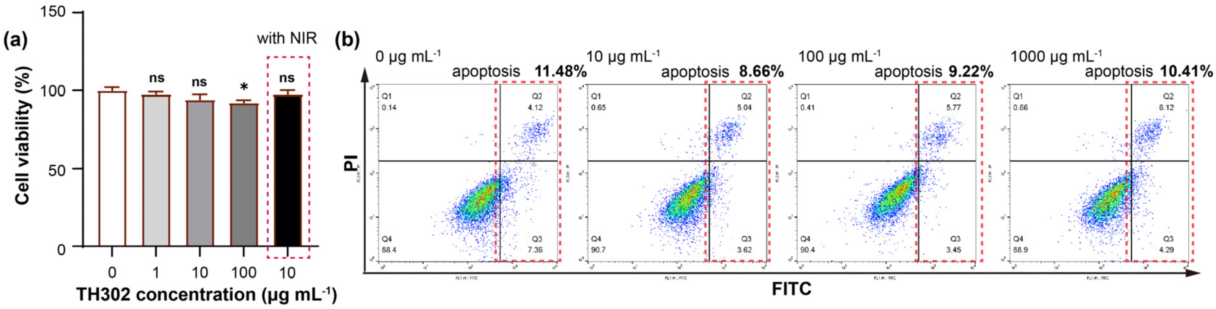

Figure S25 (a) Viability of Hep1-6 cells incubated with TH302 at various concentrations measured by CCK-8 method (n=3). (b) Apoptosis rate measured by cell flow cytometry. Values are expressed as mean ± SD. (versus N.C. group, ns: $p > 0.05$; *: $p < 0.05$; **: $p < 0.01$; *: $p < 0.001$; *: $p < 0.0001$ determined using one-way ANOVA followed by Turkey's test).

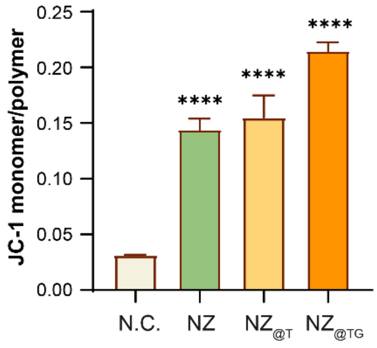

Figure S26 Quantification of JC-1 monomer/polymer ratio (n=3).

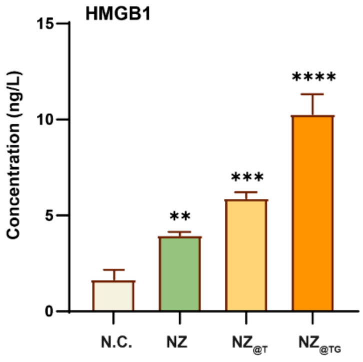

Figure S27 Quantification of HMGB1 in cell supernatant by ELISA test (n=3).

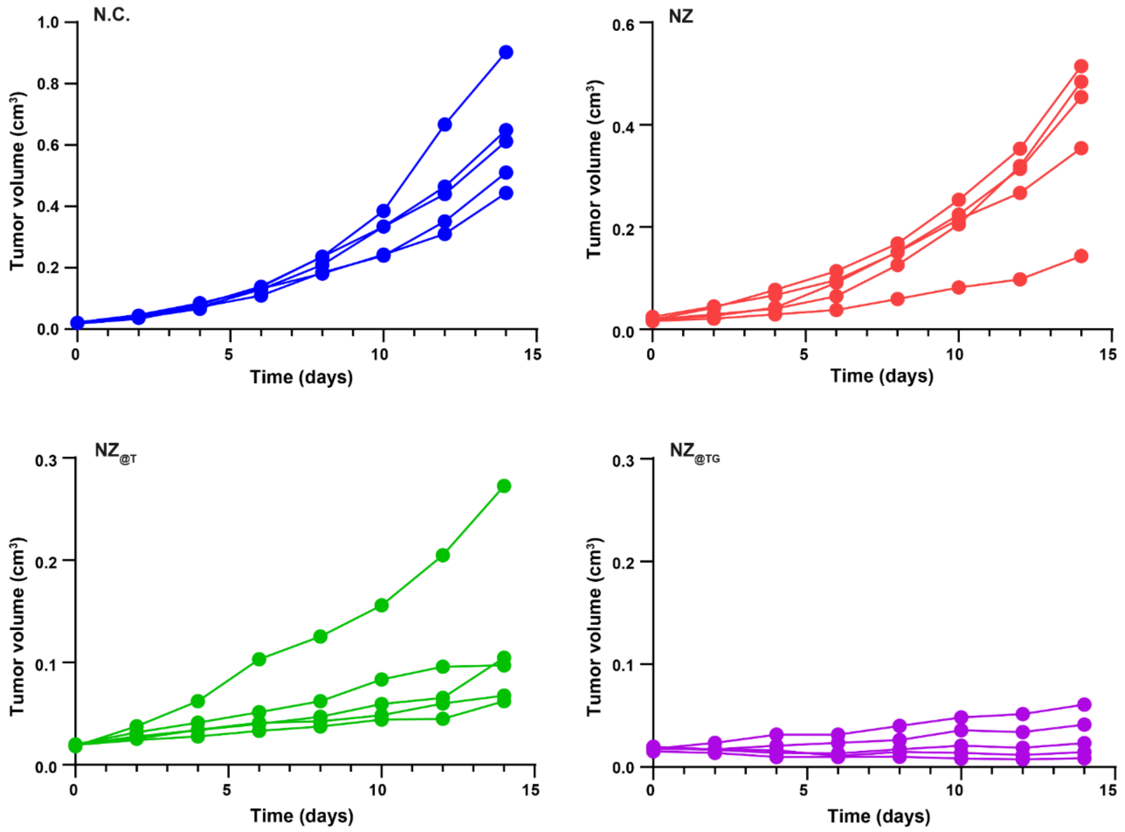

Figure S28 Tumor volume over time in different groups of subcutaneous models.

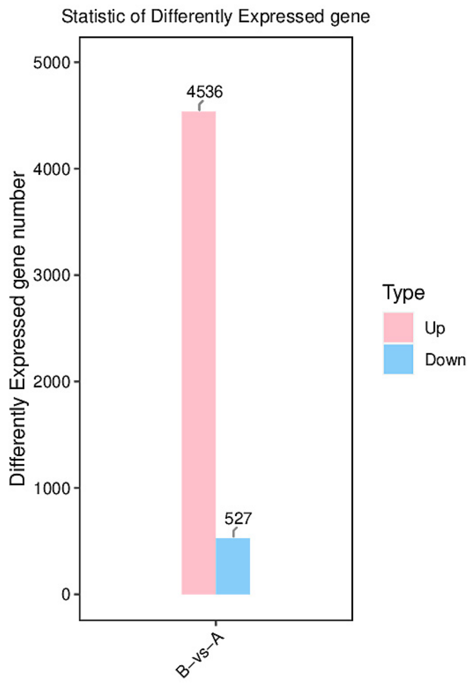

Figure S29 Numbers of upregulated and downregulated genes of the NZ@TG group in comparison to the control group.

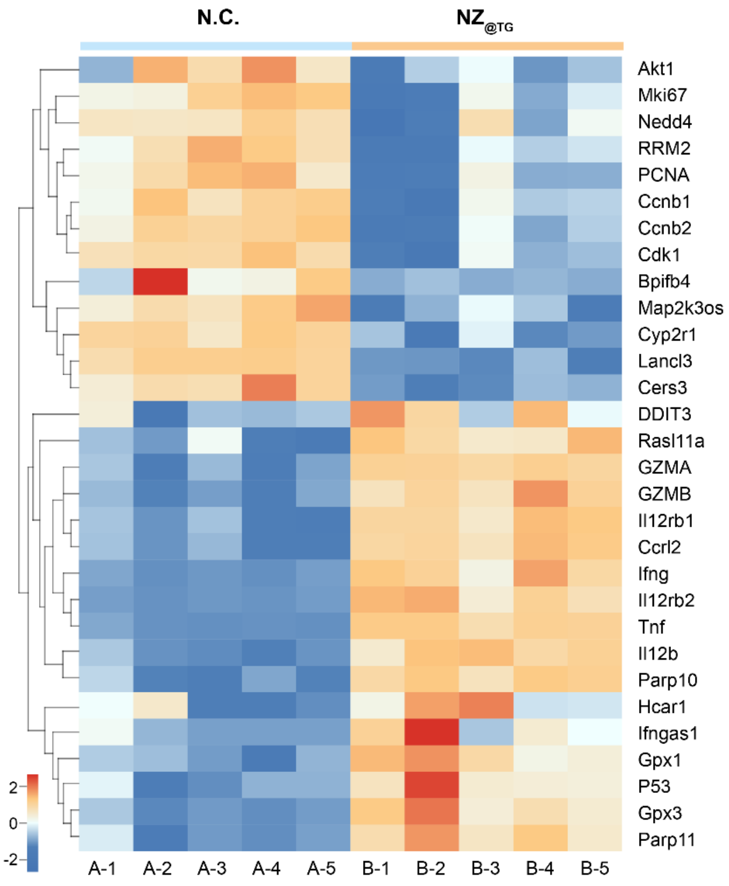

Figure S30 Heat map in pertinent to transcriptomics of subcutaneous H22 liver tumor.

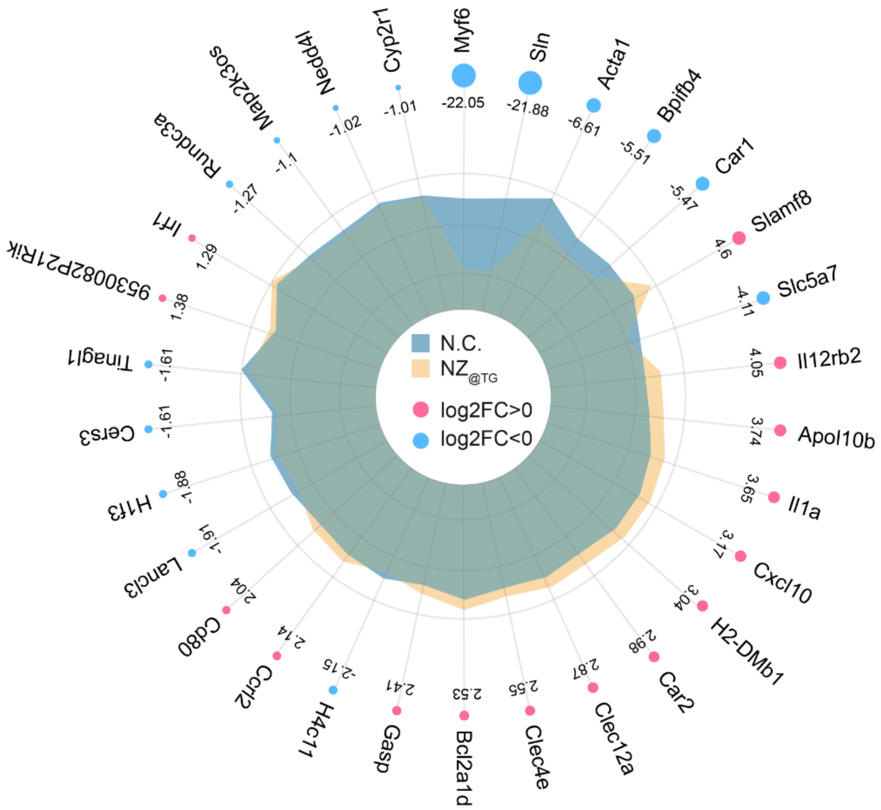

Figure S31 Radar map of differential gene expression level in subcutaneous H22 liver tumor.

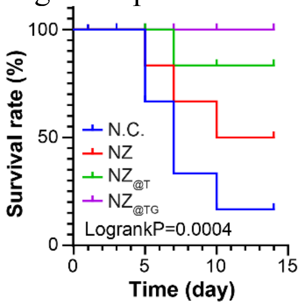

Figure S32 Survival of orthotopic liver H22 tumor bearing mice after treatment (n=6).

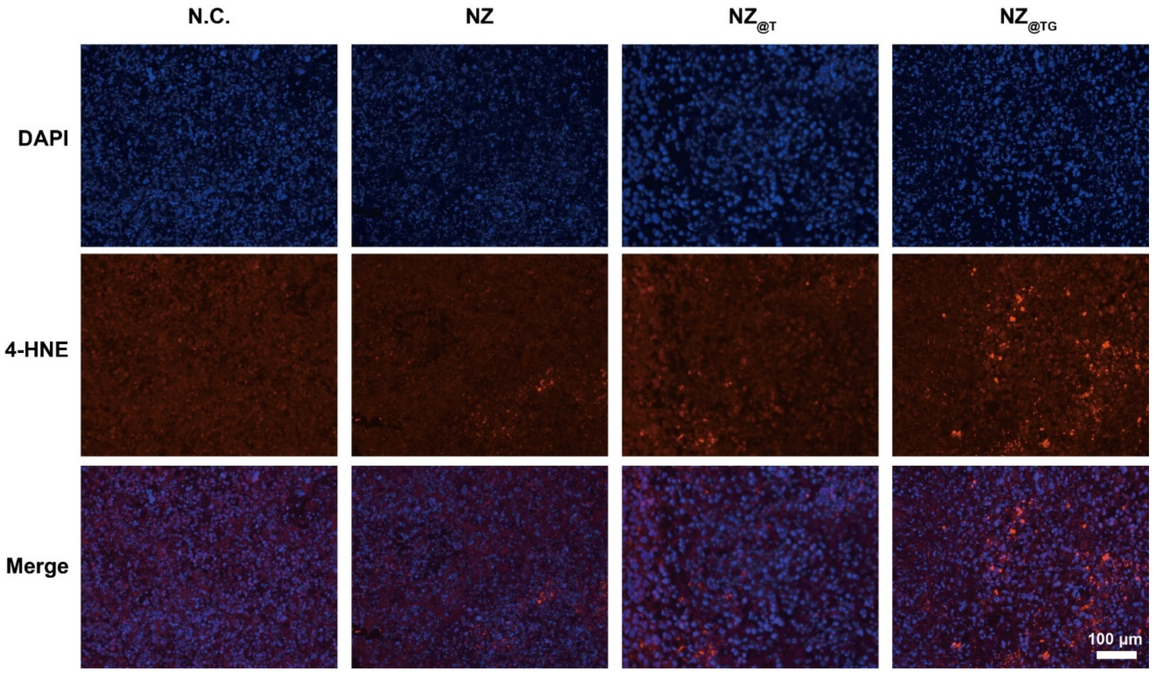

Figure S33 4-HNE staining of tissue slices from orthotopic H22 liver tumors after various treatments.

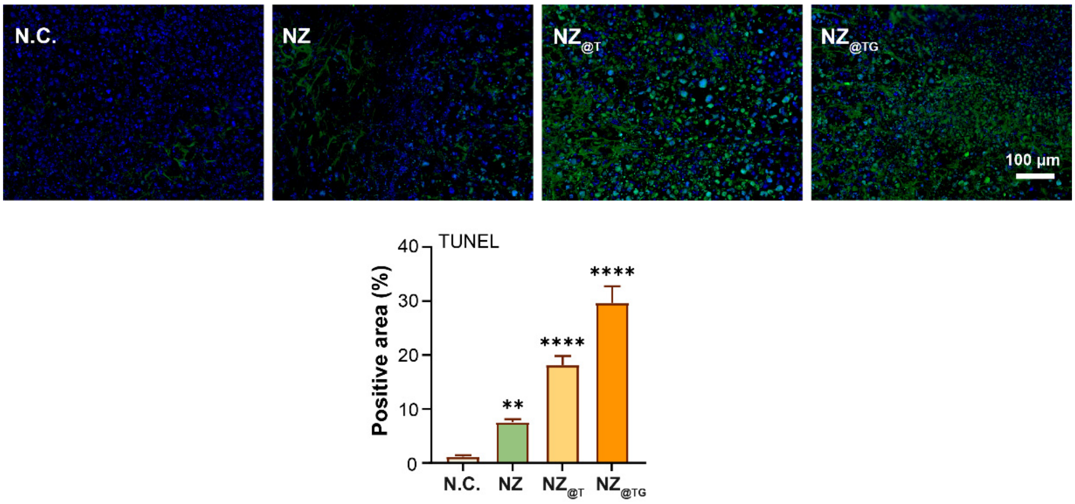

Figure S34 TUNEL staining of tissue slices from orthotopic H22 liver tumors after various treatment and the quantification results (scale bar: 100 μm).

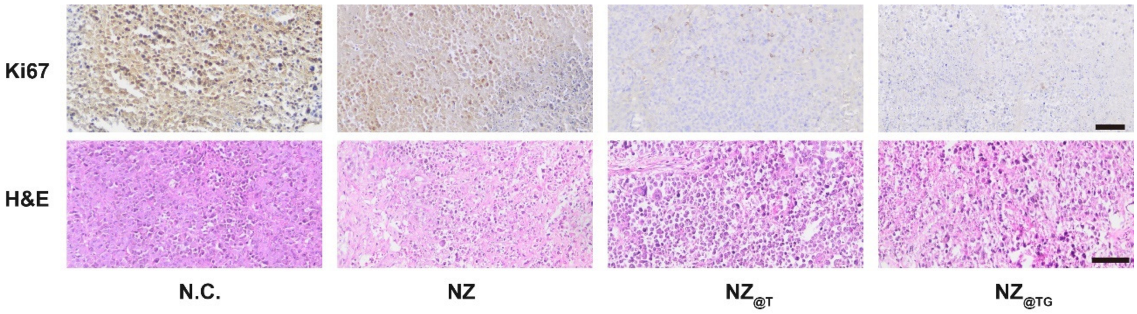

Figure S35 Ki67 and H&E staining of tissue slices from orthotopic H22 liver tumors after various treatment (scale bar: 100 μm).

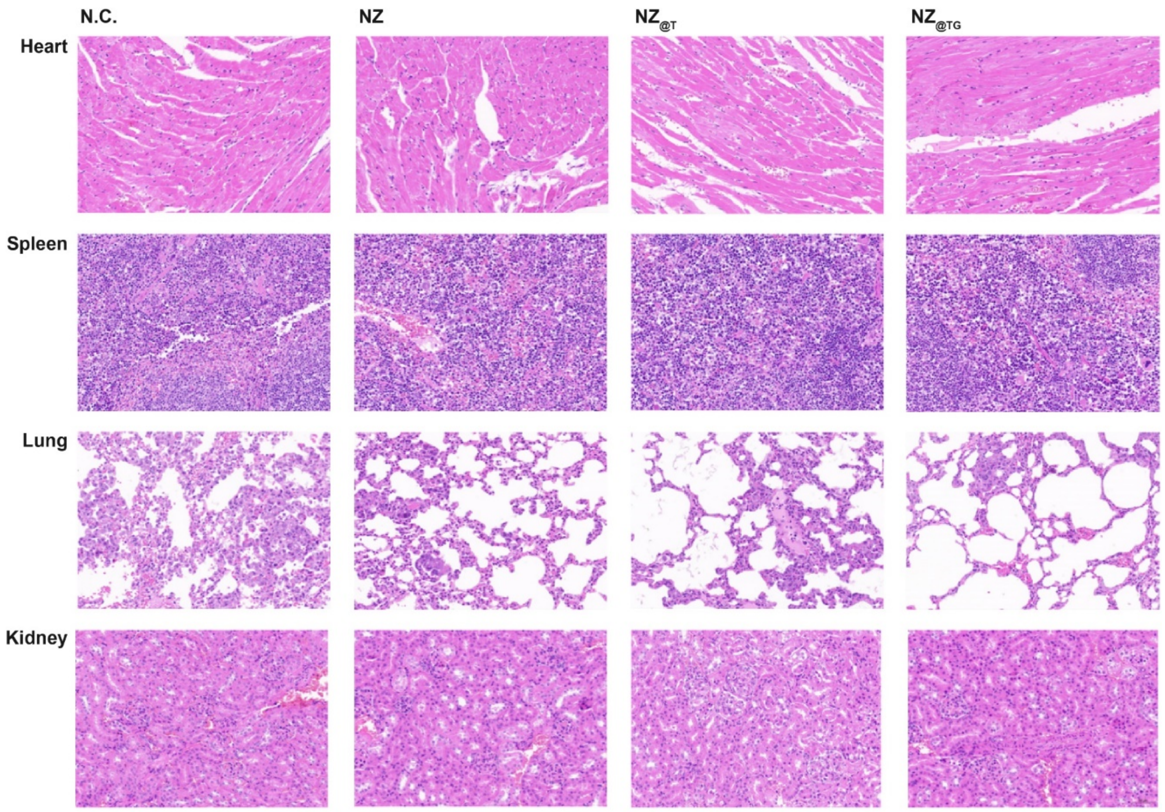

Figure S36 H&E stain of heart, spleen, lung, and kidney tissues from orthotopic H22 liver tumor bearing mice with various treatments.

Results show that phototreatment with nanocomposites did not bring about noticeable damage to important organs.

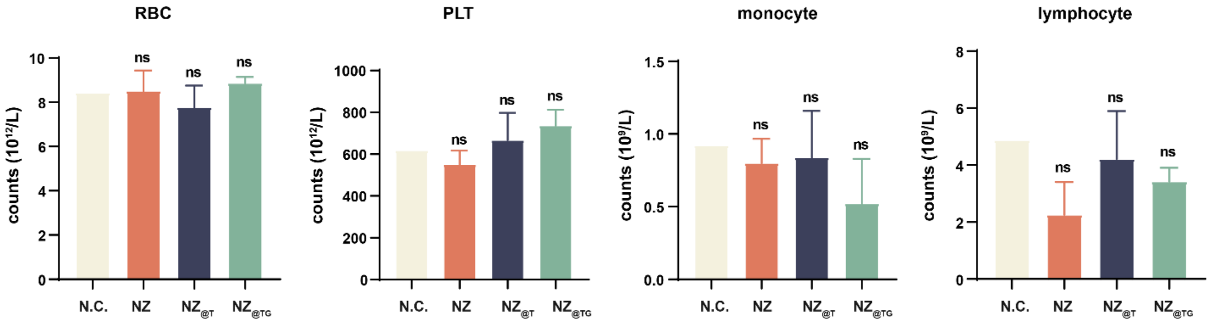

Figure S37 Blood routine examination results of orthotopic H22 liver tumor bearing mice with various treatments.

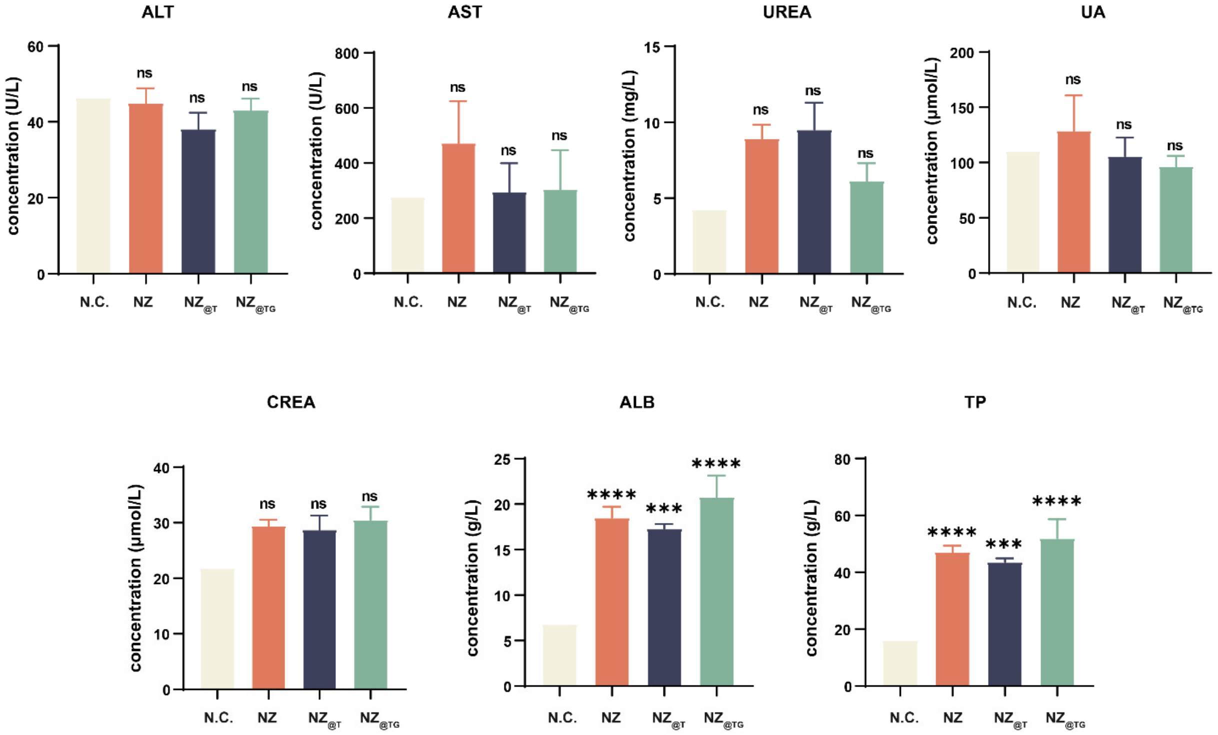

Figure S38 Blood biochemical analysis results of orthotopic H22 liver tumor bearing mice with various treatments.

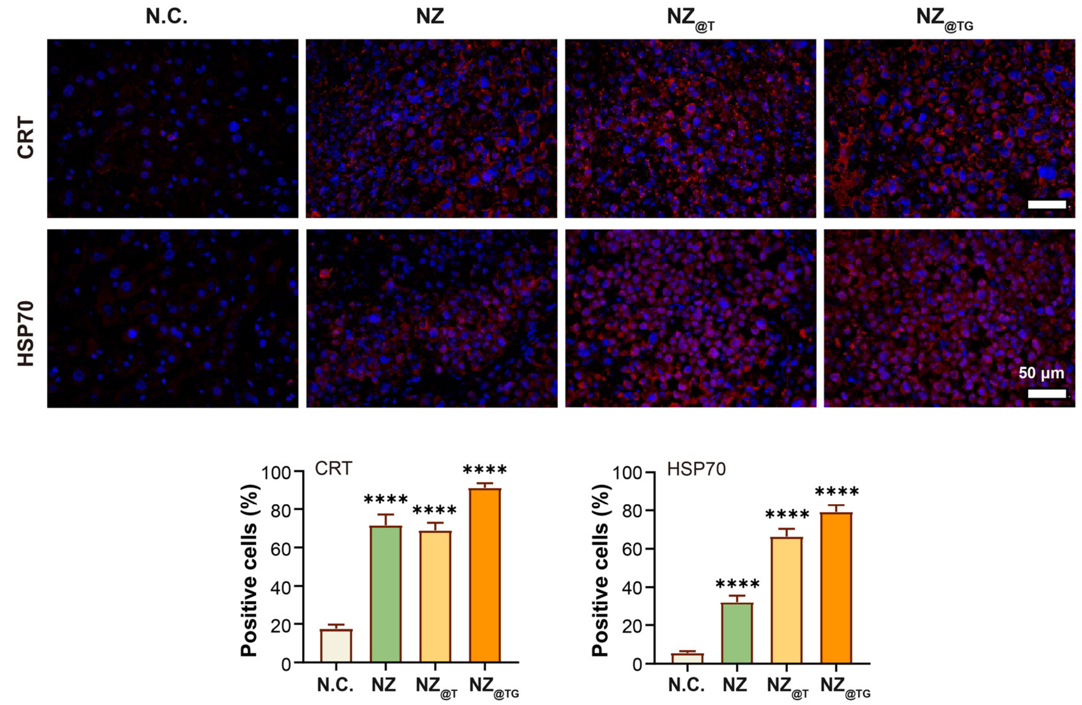

Figure S39 Immunofluorescence images and quantification results of CRT and HSP70 stained tissue slices from orthotopic H22 liver tumors after various treatments.

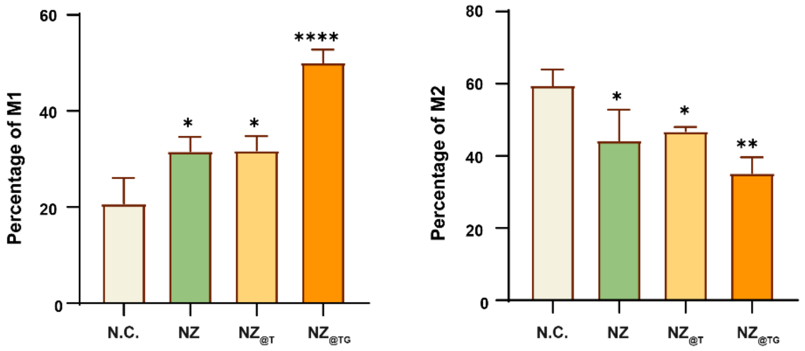

Figure S40 Quantification of percentage of M1 or M2 macrophages in orthotopic H22 liver tumors with various treatment by immune cell flow cytometry.

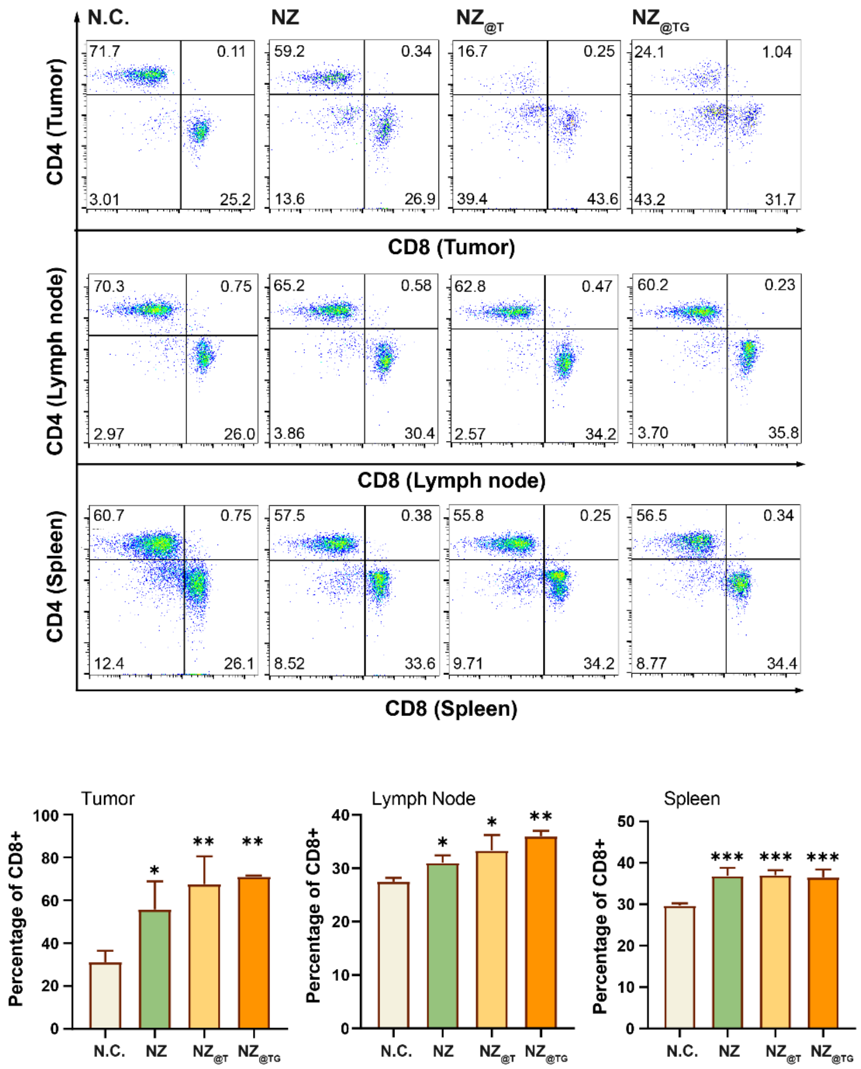

Figure S41 Immune cell flow cytometry results of spleen tissues from orthotopic H22 liver tumor models and quantification results of $CD8^+$ cells in spleen, lymph node, and tumor.

The increase of $CD8^+$ cell contents indicated the activation of systematic immune responses.

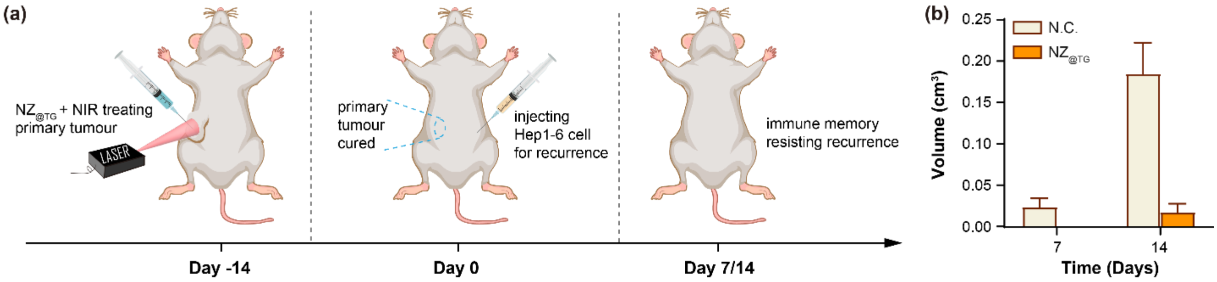

Figure S42 (**a**) Schematic illustration of the construction of recurrence tumor model after treating primary subcutaneous tumors. (**b**) Variation of recurrence tumor volume over time. Schematic illustration was created with BioGDP (https://biogdp.com/).

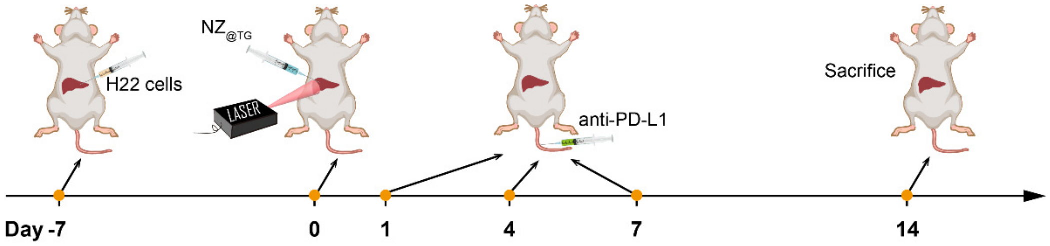

Figure S43 Therapeutic protocol of combined therapy of NIR-PDT and ICB. Schematic illustration was created with BioGDP (https://biogdp.com/).

Table S1.

Colocolization parameter	LnNP w/o GA	LnNP w/ GA
Pearson's colocolization coefficient	0.4869	0.6448
Mander's M1 (proportion of colocolized MitoRed)	0.9282	0.9305
Mander's M2 (proportion of colocolized 5-FAM)	0.7890	0.9722

Colocalization parameters calculated using ImageJ.
